# Economic evaluation of five first-line PD-(L)1 inhibitors for treating non-squamous non-small cell lung cancer in China: A cost-effectiveness analysis based on network meta-analysis

**DOI:** 10.3389/fphar.2023.1119906

**Published:** 2023-03-20

**Authors:** Xi Chen, Mingye Zhao, Lei Tian

**Affiliations:** ^1^ Department of Pharmacoeconomics, School of International Pharmaceutical Business, China Pharmaceutical University, Nanjing, China; ^2^ Center for Pharmacoeconomics and Outcomes Research, China Pharmaceutical University, Nanjing, China

**Keywords:** non-squamous non-small cell lung cancer, cost-effectiveness, partitioned survival, fractional polynomial, network meta-analysis

## Abstract

**Background and objective:** Non-small cell lung cancer (NSCLC) is one of the most malignant cancer types that causes substantial economic burden in China. This study aimed to evaluate the cost-effectiveness of five first-line anti-PD-(L)1 treatments, including sintilimab, camrelizumab, atezolizumab, pembrolizumab and sugemalimab with each combined with chemotherapy, for treating advanced non-squamous NSCLC (nsq-NSCLC) from Chinese healthcare system perspective.

**Methods:** Clinical data were obtained from the following clinical trials, namely, ORIENT-11, CameL, IMpower132, KEYNOTE-189 and GEMSTONE-302. A network meta-analysis was performed based on fractional polynomial models. We constructed a partitioned survival model with a three-week cycle length and a lifetime horizon to derive the incremental cost-effectiveness ratio (ICER). We performed one-way sensitivity analysis and probablistic sensitivity analysis to test the robustness. Additionally, two scenario analyses were undertaken to investigate the impact of Patient Assistant Program on the economic conclusion and to explore potential uncertainty associated with population representativeness of the global trial.

**Results:** Compared with camrelizumab + chemotherapy, sugemalimab + chemotherapy and atezolizumab + chemotherapy were dominated, and the ICERs generated from sintilimab + chemotherapy and pembrolizumab + chemotherapy were $15,280.83/QALY and $159,784.76/QALY, respectively. Deterministic sensitivity analysis showed that uncertainty around ICERs was mainly driven by HR related parameters derived from NMA and drug price. The probablistic sensitivity analysis suggested that camrelizumab treatment was cost-effective at a willingness-to-pay threshold of 1-time GDP *per capita*. When the threshold was set as 3-time GDP *per capita*, sintilimab strategy demonstrated the excellent cost-effective advantage. Sensitivity analysis proved the reliability of base-case results. Results from two scenario analyses indicated that the primary finding was robust.

**Conclusion:** In current context of Chinese healthcare system, sintilimab + chemotherapy appeared to be cost-effective for the treatment of nsq-NSCLC compared with sugemalimab, camrelizumab, pembrolizumab as well as atezolizumab combined with chemotherapy.

## 1 Introduction

Worldwide, lung cancer is one of the most common cancer types and is recognized to be the leading cause of cancer death (Sung et al., 2021). As statistics from International Agency for Research on Cancer revealed, newly diagnosed lung cancer cases accounted for 11.4% of total new cancer cases and 18% of total new cancer deaths were attributable to lung cancer in 2020. Non-small cell lung cancer (NSCLC) took up approximately 85% of overall lung cancer cases and the majority of NSCLC cases are classified as non-squamous NSCLC (nsq-NSCLC) (Chen et al., 2016; Duma et al., 2019). Latest statistics released by National Cancer Center showed that, in China, with both incidence rate and mortality rate of lung cancer ranking first among all cancer types, lung cancer remained the most distressing disease, resulting in almost 828,000 new cases and 657,000 deaths (Zheng et al., 2022). With the aging population, financial pressure imposed by lung cancer presents a huge challenge for health expenditure in society. A study based on claim data from China’s urban basic medical insurance estimated that, between the year of 2013 and 2016, average total medical costs incurred by lung cancer were $4,751 per patient and medicine utilization was the main factor leading to the substantial lung caner-related costs (Zhu D. et al., 2021).

More than 60% of NSCLC patients progress to clinical stage III or IV at initial diagnosis, and for those suffering from advanced stage NSCLC, prognosis is rather poor (Kocher et al., 2015). Despite that multiple chemotherapy combinations, including pemetrexed, platinum-based chemotherapy, gemcitabine, and paclitaxel exist, as were recommended to be first-line chemotherapy opitons by the National Comprehensive Cancer Network (NCCN) guideline (National Comprehensive Cancer Network, 2022), the survival benefits associated with them are far from satisfactory (Schiller et al., 2002). With therapy advances, the emergence of anti-PD-(L)1 agents transformed the paradigm of NSCLC treatment. PD-(L)1 antibodies which function *via* stimulating the immune system to capture and eliminate cancer cell are associated with superior specificity and can bring durable antitumor responses (Kaufman et al., 2019; Khozin et al., 2019). Immuno-oncology treatments encompassing sintilimab, camrelizumab, atezolizumab, pembrolizumab and sugemalimab each combined with chemotherapy have shown notable clinical effects. Based on the analysis of Chinese patients, the ORIENT-11 trial (Yang et al., 2020) reported that the median progression-free survival (mPFS) of the sintilimab group was significantly extended compared to chemotherapy group (pemetrexed + platinum) with a hazard ratio (HR) of 0.48 (95% confidence interval [CI]: 0.36–0.64). In CameL trial (Zhou et al., 2021a), the mPFS of nsq-NSCLC patients who received camrelizumab with and without chemotherapy were 11.3 and 8.3 months respectively (HR = 0.60, 95% CI: 0.45–0.79). Another trial, IMpower132 (Nishio et al., 2021), showed median overall survival (mOS) for atezolizumab combination group was 17.5 months (HR 0.86, 95% CI: 0.71–1.06). The final analysis of KEYNOTE-189 (Rodríguez-Abreu et al., 2021) reported 22.0 months of mOS for pembrolizumab + chemotherapy treatment (HR = 0.56, 95% CI: 0.46–0.69). In the lately published GEMSTONE-302 trial (Zhou et al., 2022), mPFS for sugemalimab regimen was 9.6 months (HR = 0.59, 95% CI: 0.45–0.79).

The immune checkpoint inhibitors in the abovementioned clinical trials were recommended by CSCO guideline (CSCO, 2022) for first-line treatments of nsq-NSCLC. By 2021, pembrolizumab, atezolizumab, sintilimab, camrelizumab and sugemalimab combined with chemotherapy had been successively approved for the treatment of advanced nsq-NSCLC. Sintilimab + chemotherapy and camrelizumab + chemotherapy were included in the National Drug Reimbursement List (NDRL) through price negotiation with the Chinese government in 2020 and 2021, respectively.

So far, direct comparison regarding the survival benefits across PD-(L)1 antibodies for treating nsq-NSCLC has been rather limited and the cost-effectiveness between these first-line options is unclear. Therefore, this study conducted an economic evaluation for the cost-effectiveness of these treatments in China to better inform clinical decision-making and provide cost-effectiveness evidence for the reimbursement policy.

## 2 Materials and methods

### 2.1 Target population

The population in our study was predominantly Chinese patients aged above 18 and diagnosed with nsq-NSCLC with any level of PD-(L)1 expression. In reference to characteristics of the Chinese population and related literature (Lu et al., 2017; Liu Q. et al., 2020), we assumed the average body surface area of the cohort was 1.72 m^2^ and creatinine clearance rate was 70 mL/min.

### 2.2 Interventions and comparators

To determine the treatments for our analysis, we comprehensively searched RCTs and literature published in MEDLINE, Embase, Cochrane Central Register of Controlled Trials (CENTRAL), Web of Science and ClinicalTrials.gov up to 20 October 2022 using “NSCLC,” “non-squamous,” “immune checkpoint inhibitors” and “randomized controlled trial” as key search terms. We only included studies in English without restricting the publish time. Detailed search strategies are presented in Supplementary Table S1.

Eligible studies were completed phase Ⅲ clinical trials which reported HRs, PFS and OS curves. For studies or conference abstracts based on the same RCTs, we selected the latest version. As to interventions, we focused on approved or proposed first-line combinations of PD-(L)1 blockers and chemotherapy for nsq-NSCLC patients in China. For the control group, we only included chemotherapy incorporating pemetrexed and platinum in light of comparability. Studies were selected only if PFS curves were reported and we further looked for OS curves as well as HRs. The outcomes of interest were mPFS, mOS and HR. Baseline characteristics of the populations from individual RCTs were as balanced as possible. Preferred Reporting Items for Systematic Reviews and Meta-Analysis (PRISMA) flow diagram is presented in Supplementary Figure S1.

Eventually, ORIENT-11, CameL, KEYNOTE-189, IMpower132 and GEMSTONE-302 were included and the basic information of the trials are presented in Supplementary Table S2. The trials were assessed to be at low risk of bias except two trials were not blinded (Supplementary Figure S2). To be noticed, due to the absence of survival curves of Chinese subgroup, population of KEYNOTE-189 was from around the globe. Additionally, despite the results of Chinese cohort recruited in IMpower132 has been published lately (Lu et al., 2022), we conducted base-case analysis based on the global IMpower132 trial considering its much larger sample size and longer duration of follow-up compared with those of the Chinese cohort study. Specific dosage regimens in different trials are presented in Supplementary Table S3.

### 2.3 Model design

**FIGURE 1:**
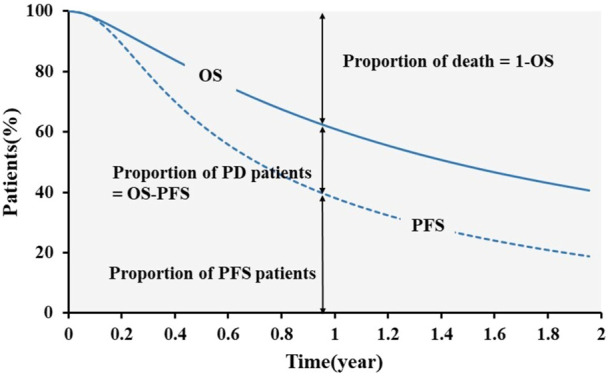
Model structure of the partitioned survival model.

For the economic evaluation, a partitioned survival model (PSM) was built with Microsoft Excel (Microsoft Inc., Redmond, Washington). The PSM incorporated three states: progression-free (PF), progressive disease (PD) and death (as shown in Figure 1). Calculation methods of the proportion of patients in any health state at specific time are as follows: the number of PFS patients was derived from the PFS curves; the proportion of PD patients was calculated as the difference between OS and PFS curves; the proportion of death was one minus the proportion of patients who were alive based on the OS curves. The time horizon was set to be lifetime which was 15 years, allowing 100% of the patients in all treatment groups to reach the state of death. According to the drug regimen, the cycle length of the model was 3 weeks. The outcomes of interest consist costs, life years, quality-adjusted life years (QALYs) and incremental cost-effectiveness ratio (ICER) expressed as the cost incurred by gaining an extra life year. Half-cycle correction following Trapezoidal rule was applied (Elbasha and Chhatwal, 2016; Wo et al., 2020). The cost and utility were discounted at a rate of 5% (Liu G. et al., 2020). Both internal and external validation approaches to confirm the model structure were conducted. With regard to internal validation, the model inputs and codes used for the analysis were checked by inviting a third reviewer to perform an independent validation. Besides, in order to investigate external validity, we searched real-world studies on PD-(L)1 inhibitors of interest for treating nsq-NSCLC, but no suitable literature could be found to validate the simulated mPFS. Thus, we compared the mPFS obtained by the model with the values reported in the RCTs and found that the model simulated well (Yang et al., 2020; Nishio et al., 2021; Rodríguez-Abreu et al., 2021; Zhou et al., 2021a; Zhou et al., 2022).

### 2.4 Clinical inputs and survival estimates

Because existing evidence of direct comparisons between the interventions are limited, we performed a network-meta analysis (NMA) to assist the evaluation of the cost-effectiveness of the five first-line immunotherapeutic options. We conducted a frequentist NMA (Rücker, 2012) to compare the effects of all treatments. Due to a lack of data to support the assessment of heterogeneity between studies, we applied the fixed-effect model (Su et al., 2020).

For survival fitting, we took the chemotherapy treatment in IMpower132 trial as the anchor because in the case of PFS maturity (Gebski et al., 2018), IMpower132 (Nishio et al., 2021) demonstrated the highest maturity (95.8%) compared to that of other trials and the duration of follow-up is relatively long. We used GetData Graph Digitizer (the version of 2.25) and applied the method established by Guyot et al. (2012), which is the generally accepted approach to recreate pseudo-individual-level data. Apart from eight standard parametric methods (weibull, exponential, gamma, log-normal, log-logistic, gompertz, generalized gamma and generalized F), fractional polynomial (FP) models (Jansen, 2011) (first order), Royston–Parmar (RP) models (Rutherford et al., 2020) (*K* = 1–5) and restricted cubic spline (RCS) models (Rutherford et al., 2020) (*K* = 1–5) were used in our study to fit the survial curves as accurately as possible. Best-fitting distributions were identified *via* Akaike information criterion (AIC) and visual inspection, along with the consideration of clinical plausibility. For instance, if the model demonstrated a low AIC value but fitted survival rates were unreasonably high and remained constant over time, the model would be identified as violating the clinical reality and would not be selected. Age- and sex-specified general population mortality which obtained from the sixth national census results in the National Bureau of Statistics (National Bureau of Statistics, 2022) were extracted to adjust the estimated OS and PFS rates.

Because the GEMSTONE-302 trial did not report the complete OS curves reflecting the survival of nsq-NSCLC patients who received sugemalimab + chemotherapy, we performed an NMA of OS based on constant HRs. We made the indirect comparison based on reported HRs by the Netmeta package in R (version of 4.2.1) assuming the homogeneity of the chemotherapy administered in control groups. The calculation equations are as follows:

$$\ln\left(HR\right)=\frac{\left[\ln\left({UL}_{HR}\right)+\ln\left({LL}_{HR}\right)\right]}{2}$$ (1)

$$seln\left(HR\right)=\frac{\left[\ln\left({UL}_{HR}\right)-\ln\left({LL}_{HR}\right)\right]}{1.96*2}$$ (2)

Ln (HR) and seln (HR) refers to the logarithm and standard error of the HR, respectively. UL and LL represents the 95% upper and lower bound.

In order to test whether the assumption of PH was reasonable for PFS (Supplementary Figure S3) in this study, log-cummulative hazard plots of all included survial curves were drawn and we found that PH did not establish (Supplementary Figure S4). Thus, methods to conduct NMAs that were not based on the PH assumption were considered. As the technical support document from NICE mentioned (Rutherford et al., 2020), the methods comprise restricted mean survival time (RMST), piecewise exponential model (PWE model), parametric survival curves, FP and spline-based model. Zhao M. Y. et al. (2022) comprehensively described the widely-adopted FP modelling approach put forward by Jansen (2011). The FP model enables the synthesis of hazard functions for NMA and first-order FP is expressed in Eq. 3. The power parameter p takes any value from −2, −1, −0.5, 0, 0.5, 1, 2 and 3, generating 8 first-order models. Then HRs at different time points were derived from Eq. 4. When p takes the value of zero, the FP model transforms into a weibull model, and when *p* = 1, the FP model is equivalent to gompertz model (Jansen, 2011). We conducted NMA in PFS state using the FP method. The abovementioned equations are presented here:

$$\mathrm{Ln}\left(h\left(t\right)\right)=\beta_{0}+\beta_{1}*t^{\wedge P},with\;t^{0}=\log\left(t\right)$$ (3)

$$\mathrm{Ln}\left(HR_{12}\right)=\mathrm{Ln}{\left(h\left(t\right)\right)}_{1}-\mathrm{Ln}{\left(h\left(t\right)\right)}_{2}=\left(\beta_{10}-\beta_{20}\right)+{\left(\beta_{11}-\beta_{21}\right)}^{*}t^{p}=d_{0}+d_{1}*t^{p}$$ (4)

### 2.5 Adverse events

We only considered grade 3 or 4 adverse events (AEs) with the reported incidence higher than 5%. Hence, anaemia, decreased white blood cell count, decreased neutrophil count and decreased platelet count were eventually included in our analysis. We assumed that all AEs took place for one time at the initiation of the simulation. The AEs and incidence rates are listed in Table 1.

**TABLE 1:** Summary of model inputs and ranges.

Model inputs	Deterministic	Minimum	Maximum	Distribution	Source
Drug acquisition cost per cycle/$					
Sintilimab	151.90	75.95	151.90	Gamma	The latest price determined during the national reimbursement negotiation
Camrelizumab	411.81	205.91	411.81	Gamma	
Atezolizumab	4613.22	2306.61	4613.22	Gamma	Current price provided from the original manufacturer
Pembrolizumab	2520.11	1260.06	2520.11	Gamma	
Sugemalimab	1740.51	870.25	1740.51	Gamma	
Carboplatin	7.28	7.28	7.74	Gamma	The median bidding price gathered across provinces in recent 1 year
Cisplatin (2 mL:10 mg)	1.31	1.24	1.31	Gamma	
Cisplatin (6 mL:30 mg)	2.69	2.69	3.93	Gamma	
Pemetrexed (200 mg)	84.27	84.26	84.28	Gamma	
Pemetrexed (500 mg)	194.77	71.13	288.83	Gamma	
Market share of carboplatin	0.74	0.59	0.89	Beta	Rui et al. (2022)
Disease management cost per time/$					
Diagnosis	2.95	1.36	4.08	Gamma	Median values collected from the medical service price lists of Beijing, Shanghai, Guangzhou, Hunan and Fujian
Nursing	4.22	3.52	4.92	Gamma	
Intravenous injection	1.41	1.36	4.08	Gamma	
Bed	7.03	4.57	9.14	Gamma	
PD-L1 testing	48.50	38.80	58.20	Gamma	Wan et al. (2020), Zhu C. et al. (2021)
Follow-up cost per time/$					
CT	50.63	40.30	60.44	Gamma	Median values collected from the medical service price lists of Beijing, Shanghai, Guangzhou, Hunan and Fujian
Routine blood test	2.81	2.18	3.27	Gamma	
Blood chemistry examination	36.29	32.59	48.89	Gamma	
Routine urine test	0.56	0.44	0.65	Gamma	
Subsequent Treatment cost per cycle/$					
Docetaxel	29.14	28.35	30.26	Gamma	Calculated from prices in the fifth Volume-based Procurement scheme, 2021
BSC	338	159	476	Gamma	Lu et al. (2017)
AE management cost/$					
Decreased neutrophil count	115.01	51.11	357.80	Gamma	Chen P. et al. (2022)
Decreased white blood cell count	115.01	51.11	357.80	Gamma	Chen P. et al. (2022)
Decreased platelet count	1505.92	1240.17	1771.67	Gamma	Chen P. et al. (2022)
Anaemia	138.75	106.73	160.10	Gamma	Chen P. et al. (2022)
End-of-life care cost/$	2298.86	892.71	6140.16	Gamma	Rui and Li (2020)
Incidence of AEs in various regimens					
Sintilimab Group					
Decreased neutrophil count	0.365	0.292	0.438	Beta	Yang et al. (2020)
Decreased white blood cell count	0.147	0.117	0.176	Beta	Yang et al. (2020)
Decreased platelet count	0.120	0.096	0.144	Beta	Yang et al. (2020)
Anaemia	0.150	0.120	0.180	Beta	Yang et al. (2020)
Camrelizumab Group					
Decreased neutrophil count	0.380	0.304	0.457	Beta	Zhou et al. (2021a)
Decreased white blood cell count	0.195	0.156	0.234	Beta	Zhou et al. (2021a)
Decreased platelet count	0.166	0.133	0.199	Beta	Zhou et al. (2021a)
Anaemia	0.185	0.148	0.222	Beta	Zhou et al. (2021a)
Pembrolizumab Group					
Decreased neutrophil count	0.163	0.130	0.196	Beta	Rodríguez-Abreu et al. (2021)
Decreased platelet count	0.084	0.067	0.101	Beta	Rodríguez-Abreu et al. (2021)
Anaemia	0.185	0.148	0.222	Beta	Rodríguez-Abreu et al. (2021)
Sugemalimab Group					
Decreased neutrophil count	0.325	0.260	0.390	Beta	Zhou et al. (2022)
Decreased white blood cell count	0.141	0.113	0.169	Beta	Zhou et al. (2022)
Decreased platelet count	0.103	0.083	0.124	Beta	Zhou et al. (2022)
Anaemia	0.134	0.108	0.161	Beta	Zhou et al. (2022)
Docetaxel					
Decreased neutrophil count	0.107	0.086	0.129	Beta	Rittmeyer et al. (2017)
Decreased white blood cell count	0.130	0.104	0.156	Beta	Rittmeyer et al. (2017)
Anaemia	0.057	0.046	0.069	Beta	Rittmeyer et al. (2017)
Duration of AEs/day					
Decreased neutrophil count	4.19	3.35	5.03	Gamma	Rui et al. (2022)
Decreased white blood cell count	4.50	3.60	5.40	Gamma	Rui et al. (2022)
Decreased platelet count	47.29	37.83	56.75	Gamma	Rui et al. (2022)
Anaemia	6.83	5.46	8.20	Gamma	Rui et al. (2022)
Survival Parameters					
HR for OS (compared with chemotherapy group)					
Sintilimab + chemotherapy	0.61	0.49	0.73	Normal	NMA
Camrelizumab + chemotherapy	0.73	0.58	0.88	Normal	
Atezolizumab + chemotherapy	0.86	0.69	1.00	Normal	
Pembrolizumab + chemotherapy	0.56	0.45	0.67	Normal	
Sugemalimab + chemotherapy	0.84	0.67	1.01	Normal	
FP parameters for PFS (compared with chemotherapy group)					
d0: Sintilimab + chemotherapy^a^	−0.46	−1.18	0.30	Uniform	NMA
d1: Sintilimab + chemotherapy^a^	−0.13	−0.44	0.18	Uniform	
d0:Camrelizumab + chemotherapy^a^	−1.11	−1.93	−0.29	Uniform	
d1:Camrelizumab + chemotherapy^a^	0.29	−0.08	0.65	Uniform	
d0: Atezolizumab + chemotherapy^a^	−0.46	−0.97	0.06	Uniform	
d1: Atezolizumab + chemotherapy^a^	−0.04	−0.26	0.18	Uniform	
d0:Pembrolizumab + chemotherapy^a^	−0.52	−0.99	−0.04	Uniform	
d1:Pembrolizumab + chemotherapy^a^	−0.10	−0.29	0.10	Uniform	
d0: Sugemalimab + chemotherapy^a^	−0.35	−1.10	0.43	Uniform	
d1: Sugemalimab + chemotherapy^a^	−0.08	−0.38	0.23	Uniform	
Proportion of patients receiving BSC in subsequent treatment					
Sintilimab group	0.524	0.419	0.629	Beta	Yang et al. (2020)
Camrelizumab group	0.420	0.336	0.504	Beta	Zhou et al. (2021a)
Atezolizumab group	0.613	0.490	0.736	Beta	Nishio et al. (2021)
Pembrolizumab group	0.505	0.404	0.606	Beta	Rodríguez-Abreu et al. (2021)
Sugemalimab group	0.559	0.447	0.671	Beta	Zhou et al. (2022)
Health utility					
Utility of PFS	0.804	0.643	0.965	Beta	Nafees et al. (2017)
Utility of PD	0.59	0.47	0.71	Beta	Rui et al. (2022)
Disutilty of decreased neutrophil count	0.20	0.16	0.24	Beta	Nafees et al. (2017)
Disutilty of decreased white blood cell count	0.20	0.16	0.24	Beta	Nafees et al. (2017)
Disutilty of decreased platelet count	0.11	0.09	0.13	Beta	Tolley et al. (2013)
Disutilty of anaemia	0.07	0.06	0.09	Beta	Wan et al. (2019)
Discount rate	0.05	0.00	0.08	Beta	Liu G. et al. (2020)

^a^ HR-related parameters, more details see Eq. 4. BSC, best supportive care; AE, adverse event; HR, hazard ratio; OS, overall survival; PFS, progression-free survival; NMA, network meta-analysis; FP, fractional polynomial.

### 2.6 Utility inputs

Health states were assigned with health utilities ranging from 0 to 1 with 0 representing death and 1 indicating perfect health. The utility of PFS was 0.804 obtained from a Chinese-based research (Nafees et al., 2017). We applied PD utility of 0.590 from an economic evaluation comparing the cost-effectiveness of sintilimab + chemotherapy and camrelizumab + chemotherapy (Rui et al., 2022) in which patient-level European Organization for Research and Treatment Quality of Life Questionnaire-Core 30 (EORTC QLQ-C30) scores in ORIENT-11 were mapped into the five-level EuroQol-5-dimension (EQ-5D-5L). The disutilities caused by the aforementioned four severe AEs were obtained from other multi-center study and related cost-effectiveness research. The details of utility parameters are displayed in Table 1.

### 2.7 Resource utilization and costs

From the perspective of healthcare system, we only considered direct medical costs. All the costs were updated to 2022 US dollars (Refinitiv, 2022) ($1 = ¥7.11).

To facilitate the modelling process, we assumed that the induction treatment period lasted for four cycles across all regimens. Each PD-(L)1 inhibitor was administered every 3 weeks and avail up to 2 years until disease progression or related severe AEs ocurred. The chemotherapy regimens included platinum (carboplatin or cisplatin) and pemetrexed. When the same drug was present in different doses, we referred to the dosage and market application following the principle of the lowest cost. In addition, as a result of the competitive market relationship between carboplatin and cisplatin, the proportions of their usage were set to be 74% and 26% (Zhou et al., 2021b). For subsequent treatments, we assumed that patients received docetaxel or best supportive care (BSC) in parallel with clinical trials. Two specifications of docetaxel were included (0.5 mL:20mg and 4 mL:80 mg) according to the fifth Volume-based Procurement scheme in 2021. Because of limited information, the proportion of patients in sintilimab group who received docetaxel was assumed to be 52.4% which was the mean value derived from other groups. Information of subsequent treatments are listed in Supplementary Table S4. Disease management costs were collected from the medical service price lists of Beijing, Shanghai, Guangzhou, Hunan and Fujian. We considered the cost of PD-L1 testing and assumed that all patients incurred the cost in the first cycle of simulation for one time (Wan et al., 2020; Zhu C. et al., 2021). Follow-up costs depended on resources used in different health states (PF and PD). End-of-life cost occurred in the last 3 months before death and was obtained from a cost-effectiveness study based on advanced NSCLC Chinese population (Rui and Li, 2020). Detailed disease management costs and follow-up costs are shown in Supplementary Table S5. We assumed that AE management costs were one-off in the modelling. Costs of AE management were based on literature and expert opinions. Key inputs related to costs are listed in Table 1.

### 2.8 Sensitivity analysis and scenario analysis

We carried out a one-way sensitivity analysis to evaluate the robustness of base-case results. The ranges of variation are illustrated in Table 1. We deemed incremental net monetary benefit (INMB) as the outcome and 1–3 times *per capita* GDP as the threshold of willingness-to-pay (WTP) (Liu G. et al., 2020). The INMB was derived from Eq. 5. The results were demonstrated in the form of tornado diagrams. Besides, we conducted a probablistic sensitivity analysis (PSA) *via* Monte Carlo simulation in which every key input was assumed to fit a theoretical distribution. The results of 10,000 iterations were drawn in the form of scatter plot and cost-effectiveness acceptance curves (CEACs). Parametric distributions and ranges used in the analysis are displayed in Table 1.

INMB=∆Utility*WTP−∆Cost (5)

In order to further explore the uncertainty of the economic results, we carried out a scenario analysis taking account of the Patient Assistance Program (PAP) for the immunotherapeutic drugs without being included in the NRDL. The detailed information of drug donation scheme is summarized in Supplementary Table S6. Apart from considering the PAP, we evaluated a second scenario in which the survival of atezolizumab arm from the Chinese cohort of IMpower132 trial (Lu et al., 2022) was incorporated in the model simulation to investigate the uncertainty potentially resulted from population representativeness of the global trial. To be specific, the PFS and OS rates of atezolizumab group from the Chinese cohort of IMpower132 trial (Lu et al., 2022) were estimated by applying the HRs assessed in the Chinese population to survival rates of the reference group in the global trial.

## 3 Results

### 3.1 Fitting and extrapolation of the survival

PFS and OS curves fitted by standard distribution models, FP, RP and RCS models are drawn in Supplementary Figures S5, S6, and the priority of these candidate models were identified. The fitting results of various models for PFS basically overlapped with the original KM curves; while for the OS stage, FP model and RCS model did not show good performance with the tails of KM curves poorly fitted. We selected the RP model (*k* = 1, Odds) and the RP model (*k* = 1, Hazard) to fit OS and PFS curves respectively considering clinical rationality and the loweset value of the AIC. AIC values derived from all candidate models are listed in Supplementary Table S7.

### 3.2 Indirect comparisons

Based on the constant HR assumption for OS, the HR generated from the pembrolizumab strategy was the lowest (0.56), followed by sintilimab, camrelizumab, sugemalimab and atezomalimab with HRs of 0.61, 0.73, 0.84 and 0.86. For the PFS stage, as illustrated in Supplementary Figure S7, when parameter p took the value of 1–3, overfitting occurred in the tail of the FP model, resulting in an unreasonable fat tail and FP models with these mentioned parameters were out of our consideration. The first order FP model (*p* = 0.5) was determined to be the best considering the lowest AIC. Table 1 displays all parameters generated from the NMA. Fitting results of FP models are shown in Supplementary Table S8.

### 3.3 Base-case analysis

In the lifetime simulation, camrelizumab therapy incurred the lowest cost ($21,527.26), followed by sintilimab ($30,295.83), sugemalimab ($71,729.38), atezolizumab ($88,022.58) and pembrolizumab ($114,669.55); pembrolizumab and sintilimab strategy yielded utilities which were close to each other and both higher than other treatments. The ICER derived from sintilimab treatment compared with camrelizumab was $15,280.83/QALY, approaching the 1-time GDP *per capita* ($11,387). When compared with sintilimab, sugemalimab and atezolizumab with each plus chemotherapy were dominated, and the ICER generated from pembrolizumab was far more than the 3-time GDP *per capita* ($34,161). Hence, sintilimab demonstrated a supremely good cost-effectiveness in the base-case analysis. The costs, life years gained, QALYs and ICERs in base-case analysis are given in Table 2.

**TABLE 2:** Base-case analysis and scenario analysis results.

Regimen	Costs	Life years	QALYs	ICER (vs. camrelizumab)	ICER (vs. sintilimab)	ICER (vs. sugemalimab)	ICER (vs. atezolizumab)
Base-case analysis results							
Camrelizumab	21,527	3.01	1.80	-	-	-	-
Sintilimab	30,296	3.51	2.37	15,281	-	-	-
Sugemalimab	71,729	2.59	1.65	Dominated	Dominated	-	-
Atezolizumab	88,023	2.53	1.60	Dominated	Dominated	Dominated	-
Pembrolizumab	114,670	3.72	2.38	159,785	9,276,566	59,236	34,332
Scenario analysis 1 results							
Camrelizumab	21,527	3.01	1.80	-	-	-	-
Sintilimab	30,296	3.51	2.37	15,281	-	-	-
Sugemalimab	30,697	2.59	1.65	Dominated	Dominated	-	-
Atezolizumab	61,183	2.53	1.60	Dominated	Dominated	Dominated	-
Pembrolizumab	73,032	3.72	2.38	88,357	4,698,729	58,403	15,267
Scenario analysis 2 results							
Camrelizumab	21,527	3.01	1.80	-	-	-	-
Sintilimab	30,296	3.51	2.37	15,281	-	-	-
Sugemalimab	71,729	2.59	1.65	Dominated	Dominated	-	-
Atezolizumab	77,517	3.10	1.86	933,632	Dominated	28,660	-
Pembrolizumab	114,670	3.72	2.38	159,785	9,276,566	59,236	71,044

QALY, quality-adjusted life years; ICER, incremental cost-effectiveness ratio.

### 3.4 Sensitivity analysis

**FIGURE 2:**
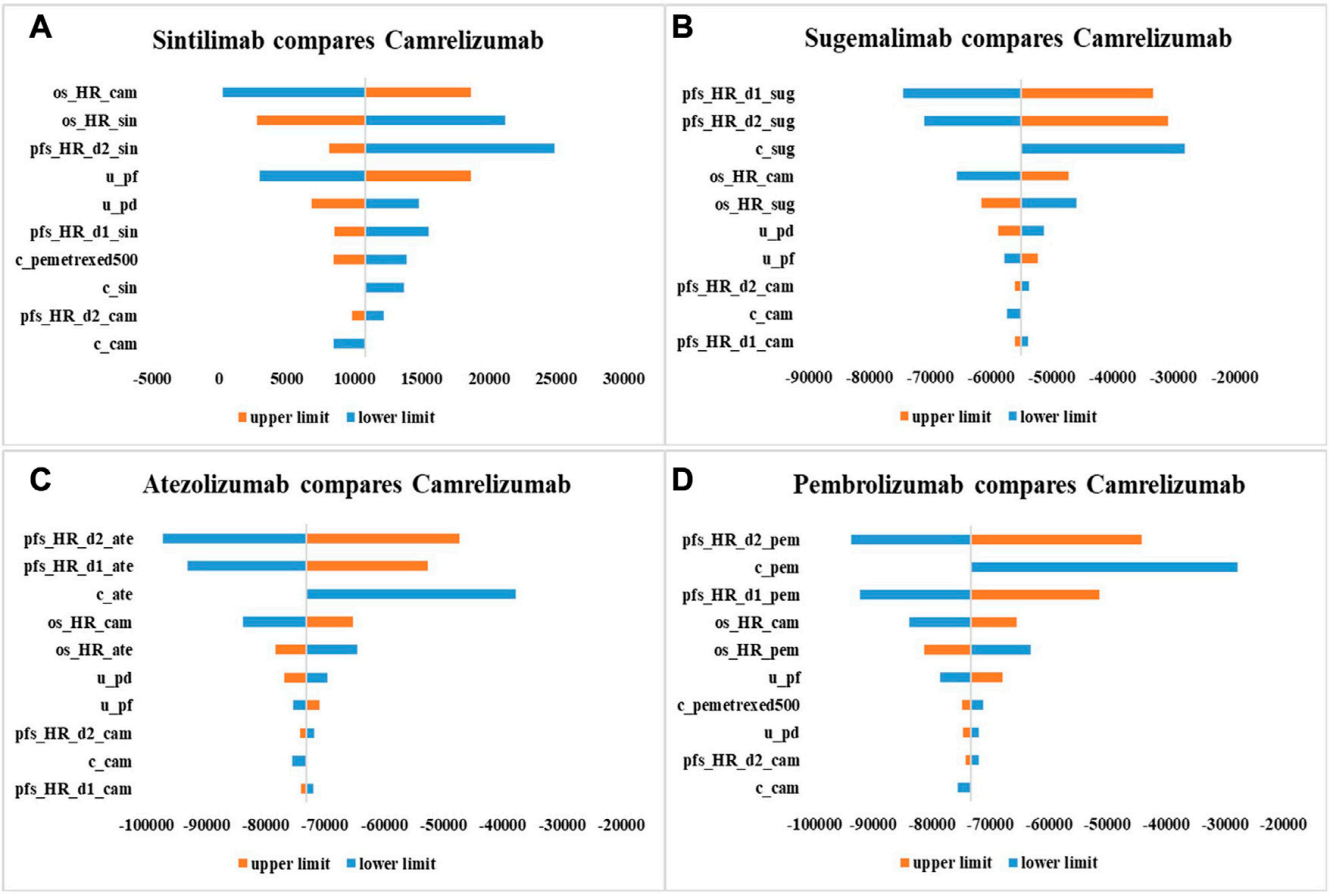
One-way sensitivity analysis results with 10,000 iterations in the form of tornado diagram. c_sin, cost of sintilimab; c_cam, cost of camrelizumab; c_sug, cost of sugemalimab; c_ate, cost of atezolizumab; c_pem, cost of pembrolizumab; c_bsc, cost of best supportive care; c_pemetrexed500, cost of pemetrexed (500 mg); u_pf, health utility of progression-free survival state; u_pd, health utility of progressive disease state; r_bsc_cam, the proportion of patients who received camrelizumab strategy and best supportive care in subsequent treatment; r_bsc_pembrolizumab, the proportion of patients who received pembrolizumab strategy and best supportive care in subsequent treatment; pfs_HR_d0_cam, pfs_HR_d1_cam: parameters for PFS HR (camrelizumab + chemotherapy vs. chemotherapy); pfs_HR_d0_sin, pfs_HR_d1_sin: parameters for PFS HR (sintilimab + chemotherapy vs. chemotherapy); pfs_HR_d0_sug, pfs_HR_d1_sug: parameters for PFS HR (sugemalimab + chemotherapy vs. chemotherapy); pfs_HR_d0_ate, pfs_HR_d1_ate: parameters for PFS HR (atezolizumab + chemotherapy vs. chemotherapy); pfs_HR_d0_pem, pfs_HR_d1_pem: parameters for PFS HR (pembrolizumab + chemotherapy vs. chemotherapy); os_HR_cam, OS HR (camrelizumab + chemotherapy vs. chemotherapy); os_HR_sin, OS HR (sintilimab + chemotherapy vs. chemotherapy); os_HR_sug, OS HR (sugemalimab + chemotherapy vs. chemotherapy); os_HR_ate, OS HR (atezolizumab + chemotherapy vs. chemotherapy); os_HR_pem, OS HR (pembrolizumab + chemotherapy vs. chemotherapy).

As shown by tornado diagrams (Figures 2A–D), the INMBs generated from combination therapy linked with sugemalimab, atezolizumab and pembrolizumab were less than 0, implying that these options were less cost-effective compared with camrelizumab therapy in one-way sensitivity analysis, which was in parallel with base-case results. Under a threshold of 1–3 times GDP *per capita*, HRs for PFS and OS and drug price were the top influential factors for the INMBs. Compared with camrelizumab regimen, HR of sintilimab combination treatment *versus* chemotherapy for OS derived from the NMA imposed the greatest uncertainty to the economic results. For other regimens, the cost-effectiveness would be improved if HRs for PFS changed and prices dropped.

**FIGURE 3:**
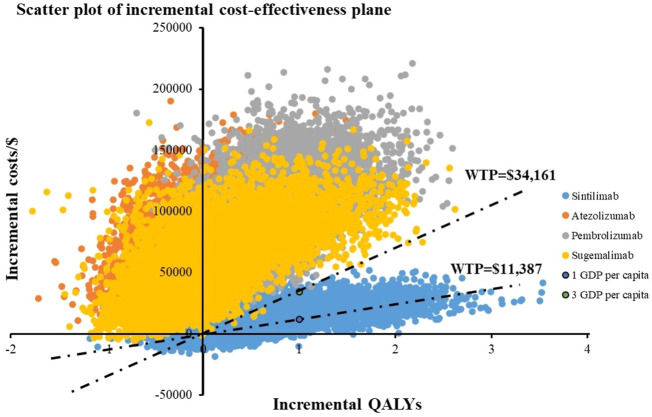
Cost-effectiveness scatter plot results with 10,000 iterations. WTP, willingness-to-pay.

**FIGURE 4:**
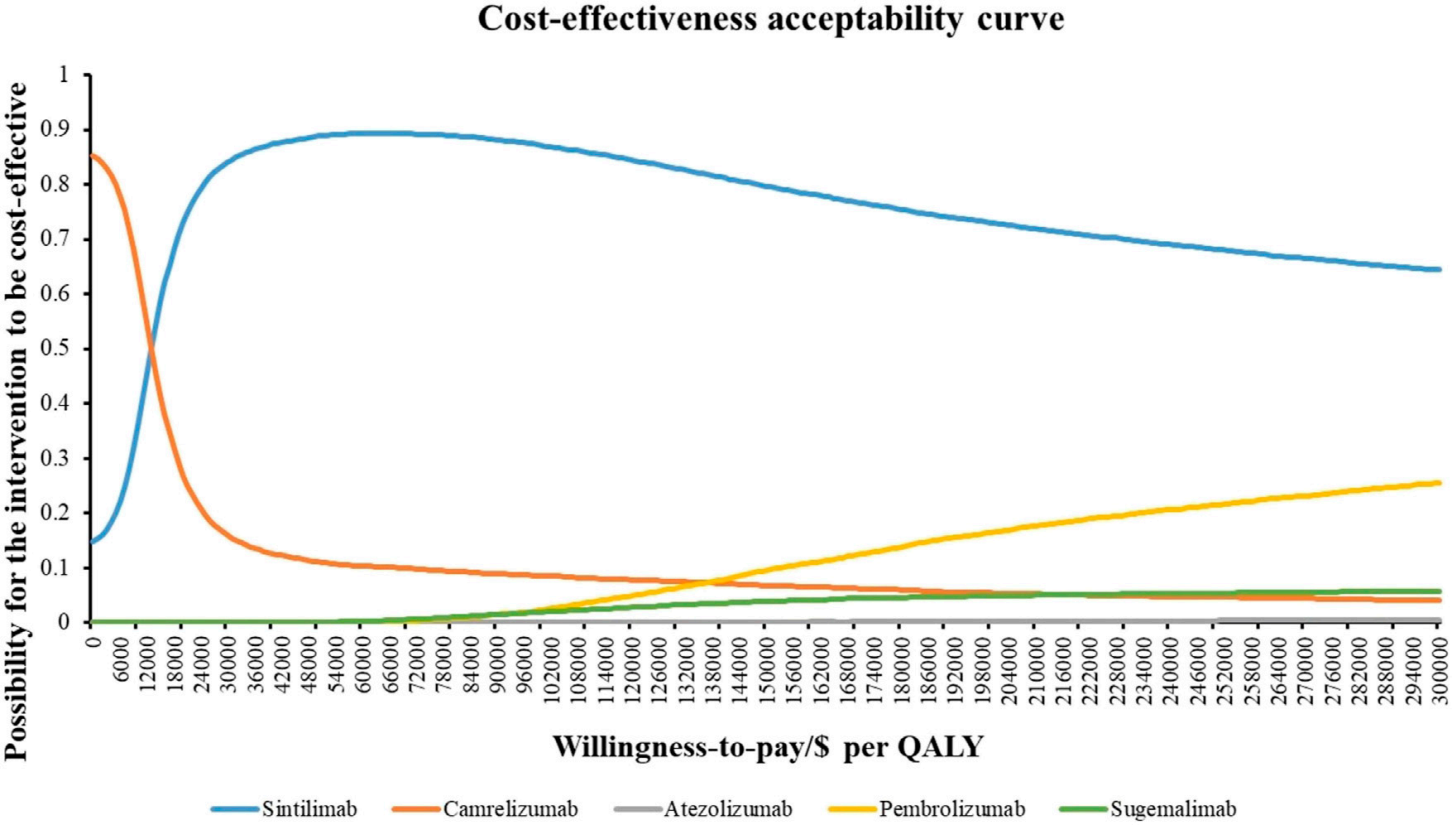
Cost-effectiveness acceptability curves of base-case probablistic sensitivity analysis.

The results of PSA are depicted in cost-effectiveness scatter plot (Figure 3) and CEACs (Figure 4). The scatters which represented the ICERs of sugemalimab, pembrolizumab and atezolizumab options were almost all above the three-time GDP *per capita*, meaning the probability for them to be cost-effective when compared with camrelizumab regimen was nearly zero percent. For sintilimab strategy, the probability of it to be cost-effective when compared with camrelizumab treatment under one time GDP *per capita* was 29.41% and 76.83% under 3-time GDP *per capita*. When WTP varied from $11,387-$13,000 per QALY gained, camrelizumab was most likely to be cost-effective; when WTP varied from $13,000 to three times *per capita* GDP ($34,161), the probability for sintilimab to demonstrate the best cost-effectiveness was the highest. Aligned with the advantage displayed in base-case analysis, sintilimab treatment appeared to have the greatest potential to be cost-effective in PSA.

### 3.5 Scenario analysis

In the first scenario, we evaluated the influence of current PAPs on study results. The acquisition costs of sugemalimab, pembrolizumab and atezolizumab were lowered with the support of PAP. Under this circumstance, the ICER of pembrolizumab regimen compared with camrelizumab was $88,356.72/QALY; sugemalimab and atezolizumab treatments were still dominated by camrelizumab strategy. In the second scenario, we explored the stability of economic results by incorporating the survival of Chinese patients from the IMpower132 to the model. With less cost required and more utilities generated, the ICER of atezolizumab compared with sugemalimab was improved, nevertheless, the cost-effectiveness of atezolizumab remained to be poor when compared with camrelizumab and sintilimab. Overall, the scenario analysis results were consistent with the base-case results. Scenario analysis results are displayed in Table 2.

## 4 Discussion

This is the first study to investigate the cost-effectiveness of sintilimab, camrelizumab, atezolizumab, pembrolizumab and sugemalimab with each in combination with chemotherapy for first-line treatment of nsq-NSCLC in China. Our findings serve to facilitate health resource allocation and provide evidence for clinical decision-making.

Due to the lack of head-to-head clinical effect comparison, we set an anchor for indirect comparison, applied a blend of models to fit the long-term survival and avoided assuming the PH which might not establish in reality. The regimens of interest were indirectly linked *via* NMA adopting first-order FP models. The principal finding of base-case analysis was that sintilimab therapy generated an advantageous ICER which approached the one-time GDP *per capita*, indicating the excellent cost-effectiveness. ICERs associated with pembrolizumab transcended three times GDP *per capita*; sugemalimab and atezolizumab were dominated when compared to camrelizumab, and hence, these three treatments did not show good cost-effectiveness in treating nsq-NSCLC in China. One-way sensitivity analysis indicated that in every comparison with camrelizumab, HRs for PFS and OS and drug cost imposed considerable uncertainty upon the economic outcome. The PSA confirmed the robustness of base-case analysis results. Scenario analysis found that although reduction in the drug prices greatly influenced the ICERs, options that were not covered in the NRDL, still, were less competitive than sintilimab therapy. When survival of Chinese population was considered in the model, the primary finding that sintilimab and camrelizumab demonstrated better cost-effectiveness remained stable.

As growing technical advances are made in immunotherapy for treating cancers, the concern for anti-PD-(L)1 therapy grows and an increasing number of studies evaluating the cost-effectiveness of anti-PD-(L)1 agents have emerged in recent 3 years. Published economic evaluations mainly based on survival data reported in single RCTs. Regarding the comparison between camrelizumab and chemotherapy, Zhu C. et al. (2021), obtained clinical information from the CameL trial and argued that camrelizumab was economically advantageous compared with chemotherapy. However, another two studies (Xiang et al., 2021; Chen T. et al., 2022), based on CameL trial as well, did not present the favorable economic outcome for camrelizumab. Yang et al. (2021) undertook the economic evaluation of atezolizumab treatment by buiding three-state Markov model and results showed that atezolizumab lacked economic advantage compared to chemotherapy. The study carried out by (Cai et al., 2021) stated that pembrolizumab, on account of philanthropic PAP, still could hardly demonstrate the possibility to be cost-effective under the suggested WTP. So far, only one study (Rui et al., 2022) has investigated cost-effectiveness compared between PD-(L)1 inhibitors in first-line treatment of advanced nsq-NSCLC. Rui et al. (2022) made direct comparison between sintilimab and camrelizumab strategy *via* constructing PSM from the Chinese healthcare system perspective, and concluded that sintilimab regimen possessed advantageous cost-effectiveness over camrelizumab due to more QALYs gained with lower costs required, which mirrored our study result.

The standard distribution models simulating the long-term survival of patients diagnosed with cancer have been widely applied in economic evaluations on immuno-oncology therapies. A majority of previous studies (Liu et al., 2021; Teng et al., 2021; Kang et al., 2022) which undertook indirect comparison overlooked the issue of PH and performed NMA by assuming the HRs remain constant as time went by, calculating survival data in treatment groups depending on the constant HR. However, with breakthroughs in immunotherapy, the survival of cancer patients probably have changed and the hazard function is foreseenably becoming more complex. According to the supportive document from NICE (Rutherford et al., 2020), standard parametric models no longer work so long as the monotonically decreasing or increasing hazards of survival no longer establish. Therefore, merely considering standard models is not reasonable. It is necessary to break the limitation of standard parametric model and free the assumption of PH in NMA. FP models have demonstrated its flexibility and plausibility in economic evaluations based on NMA and have been adopted in several studies to derive parameters essential for the time-varying HRs (Wang et al., 2021; Wang et al., 2022; Zhao M. et al., 2022). In our study, apart from applying standard parametric models for the long-term survival fitting, we incorporated more flexible parametric models, including FP, RP and RCS models which are independent of the PH assumption and the accuracy of reflecting real clinical effects were enhanced. We further provided elaborations of multiple modelling methods within our consideration as explained in Supplementary Method S1.

To be noted, our study is subject to several assumptions which may generate bias or uncertainty to the explanation of study outcomes. To begin with, the economic results were based on indirect comparison of multiple RCTs and the differences between RCTs may introduce bias to the study conclusion. Secondly, the actual effectiveness of chemotherapies across different RCTs may not resemble each other but in order to facilitate the indirect comparison, we assumed homogeneity existed in the effects. The reliability of our study results will be tested if head-to-head RCTs are conducted in the future. Additionally, we had to rely on the PH assumption for the comparison of OS across studies because the OS curve of sugemalimab was not available. As a result, bias might be introduced in the calculation of the cummulative health benefits. Besides, due to the absence of detailed information about patients who accepted active therapy or BSC in sintilimab trial, we calculated the subsequent treatment proportion from the average value of other four regimens.

## 5 Conclusion

This economic evaluation revealed that sintilimab plus chemotherapy appeared to be the superior treatment in terms of cost-effectiveness for Chinese patients diagnosed with advanced nsq-NSCLC with no prior treatment. If the WTP increases or the drug price drops, the cost-effectiveness of atezolizumab, pembrolizumab and sugemalimab strategy will be improved.

## Data Availability

The original contributions presented in the study are included in the article/Supplementary Material, further inquiries can be directed to the corresponding author.
