# Supplementary material for: Economic evaluation of five first-line PD-(L)1 inhibitors for treating non-squamous non-small cell lung cancer in China: A cost-effectiveness analysis based on network meta-analysis
Source: Front Pharmacol. 2023 Mar 20;14:1119906. doi: 10.3389/fphar.2023.1119906 (PMC10067912; doi:10.3389/fphar.2023.1119906)
Supplement: Supplementary file 1 [file Table1.DOCX]

***Supplementary Material***

**Economic Evaluation of Five First-line PD-(L)1 Inhibitors for Treating Non-squamous Non-small Cell Lung Cancer in China: A Cost-Effectiveness Analysis Based on Network Meta-Analysis**

**Xi Chen^1,2^, Mingye Zhao^1,2^, Lei Tian^1,2*^**

*** Correspondence:** Lei Tian: [cputianlei@163.com](mailto:cputianlei@163.com)

Contents

[Method S1 Model introduction 2](#_Toc128218264)

[Table S1 Details of search strategy 6](#_Toc128218265)

[Table S2 Basic information of included randomized controlled trials 8](#_Toc128218266)

[Table S3 Descriptions of treatment regimens 10](#_Toc128218267)

[Table S4 The proportion of patients receiving follow-up active treatment and best supportive care in subsequent treatment 12](#_Toc128218268)

[Table S5 Summary of disease management costs and follow-up costs 12](#_Toc128218269)

[Table S6 Information of patient assistant programs 13](#_Toc128218270)

[Table S7 The goodness-of-fit results of various models 13](#_Toc128218271)

[Table S8 AIC values of various FP models 14](#_Toc128218272)

[Figure S1 The PRISMA flow diagram 15](#_Toc128218273)

[Figure S2 Risk of bias assessment outcome 16](#_Toc128218274)

[Figure S3 Original Kaplan-Meier plots of progression-free survival 17](#_Toc128218275)

[Figure S4 Log-cummulative hazard plots of the progression-free survival of pateints diagnosed with nsq-NSCLC 18](#_Toc128218276)

[Figure S5 The fitting performance of all candidate models for progression-free survival 19](#_Toc128218277)

[Figure S6 The fitting performance of all candidate models for overall survival 19](#_Toc128218278)

[Figure S7 Fitted curves of all FP models 20](#_Toc128218279)

[Reference 21](#_Toc128218280)

# Method S1 Model introduction

Before determining the fitted model, the plots of Nelson-Aalen estimate of the log cumulative hazard was used as test for assumption of PH assumption. When the PH assumption is not established, Non-PH models should be used.

1. Cox-PH model

Cox proportional hazards model uses the HR calculated by the Cox regression model as the comparative effect size under the assumption of PH, and uses the survival rate of the bridge treatment program to calculate the survival rate of other programs. Under the Bayesian framework, a two-step method can usually be used, which is calculated as follows:

$$h_{j,bq}(t)=h_{0j,bq}(t)exp(\alpha_{j,bq}x_{\mathrm{ij}})$$

The second step is the calculation of $\hat{\alpha}_{j,bq}$. Under the Bayesian fixed effects model, $\hat{\alpha}_{j,bq}$ is the estimation of $\alpha_{j,bq}$, $Var(\hat{\alpha}_{j,bq})$ is the variance, and $\hat{\alpha}_{j,bq}$ and its confidence interval are estimated as follows:

$$\hat{\alpha}_{j,bq}\sim N(\alpha_{j,bq},Var(\hat{\alpha}_{j,bq}))$$

Non-informative normal prior distribution pairs were fitted with mean 0 and standard error 0.0001.

The HR directly reported in the RCT can also be used, and the calculation of the relative effect size is relatively simple, as follows:

$$h_{j,bq}(t)=h_{0j,bq}(t)exp(\mathrm{HR}_{\mathrm{bq}})$$

1. Piecewise Exponential Model

Piecewise exponential model assumes that HRs between treatment obey the PH assumption at different times. The general practice is to find the time point t that satisfies the piecewise PH assumption by plotting the Log cumulative risk plot. And with this point as an interval, it is assumed that the HR of the NMA model obeys an exponential distribution between 0 and t, and obeys another exponential distribution beyond time t.

The Poisson distribution can be used as the link function. $e_{ijk}$ represents the occurrence of events in patient i in each time interval k in trial j. For q+1 treatment, assuming that the PH assumption is true during time period k, the fitting process is as follows:

$$e_{ijk}\sim Poisson(\lambda_{ijk})$$

$$ln(\lambda_{\mathrm{ijk}})=\alpha_{1}\mathrm{trt}_{1jk}+...+\alpha_{q}\mathrm{trt}_{\mathrm{qjk}}+\beta_{\mathrm{jk}}+\rho_{1}\mathrm{trt}_{1jk}\omega_{k}+...+\rho_{q}\mathrm{trt}_{\mathrm{qjk}}\omega_{k}+ln(y_{\mathrm{ijk}})$$

In the above formula, $\lambda_{ijk}$ represents the hazard rate of patients in each trial within each time interval. $\mathrm{trt}_{i\mathrm{jk}}$ represents q+1 treatment, $\alpha_{i}$ represents the relative risk rate of each treatment compared to the reference regimen in the k period; $\beta_{\mathrm{jk}}$ represents the basic risk rate of the reference regimen; $\rho_{i}$ represents the change value of each regimen compared to the risk rate; $\omega_{k}$ is equal to 1 when $t>k$, otherwise equal to 0; $y_{\mathrm{ijk}}$ is the survival time limit during time period k of all patients in j trial, used as the intercept. $\beta$ and $\rho$ are used the uninformative Normal prior distribution with a mean of 0 and standard errors of 0.0001 and 0.1, respectively.

In addition, by changing the link function, the piecewise exponential model can also be transformed into piecewise Weibull, piecewise Gompertz model, etc.

1. Parametric Survival Curve Model

The core theory of parametric survival curve model is to calculate the parameter difference by modeling the shape and scale parameters of the parametric model, and on this basis, determine the risk value equation of the parametric model and the change of survival rate. Generally, the parametric models that can be considered by the parametric survival curve NMA include: exponential model, Weibull model, Gompertz model, Log-logistics and Log-normal model. The parameterization process for exponential models is relatively simple, as there is only one parameter. Other parametric models include shape parameters $(\gamma)$ and scale parameters ($\lambda$). By reparameterizing $\gamma$ and $\lambda$ to construct a corresponding risk function, the parametric equations of time-varying HR and survival rate can be obtained. The reparameterization process, risk function and survival function of each parameter model are shown in the following table:


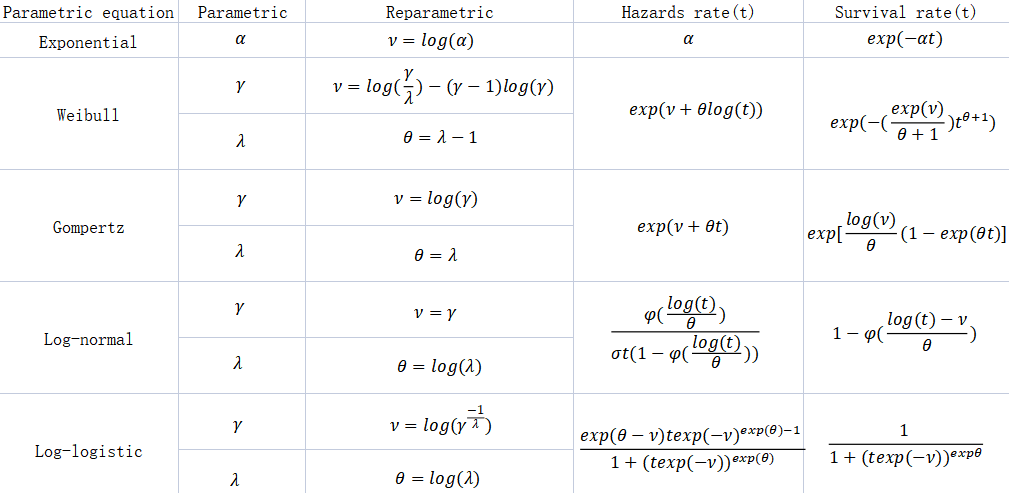


As shown in the table, the re-parameterized parameters and after $\gamma$ and $\lambda$ are obtained. The calculation of subsequent HR and survival rate are as follows:

$$\log(h(t))=\nu+\theta*log(t)$$

$$log(\mathrm{HR}_{\mathrm{AB}})=\log({h(t)}_{A})-\log({h(t)}_{B})=(\nu_{A}-\nu_{B})+(\theta_{A}-\theta_{B})\log(t)$$

$$log(\mathrm{HR}_{\mathrm{BC}})=log(\mathrm{HR}_{\mathrm{AC}})-log(\mathrm{HR}_{\mathrm{AB}})$$

$$S(t)=e^{-ln(H(t))}$$

Assuming ${d_{0}=\nu}_{A}-\nu_{B}$、${d_{1}=\theta}_{A}-\theta_{B}$, the risk rate equation formed by the parametric survival curve model can be simplified as:

$$ln(\mathrm{HR}_{t})=d_{0}+d_{1}t$$

1. Gengamma Model

Generalized gamma models can be fitted in a Bayesian framework using a two-step approach. The first step was to fit each set of study protocols into a generalized gamma model to obtain estimates and standard errors of hazard ratios. At present, three parameters can be reparameterized in the Flexsurv package. The reparameterization process is as follows:

$$f(t|\alpha,\eta,Q)=\left\{ \begin{aligned} \frac{\gamma^{\gamma}\exp(z\gamma^{-2}-u)}{\eta t\gamma^{-2}\tau(\gamma)} if Q\neq0 \\ \frac{\exp(\frac{-z^{2}}{2})}{\eta t{(2\pi)}^{-2}} if Q=0 \end{aligned} \right.$$

In the above formula, t is the survival time, $\alpha$ is the location parameter, and $\eta$ is the scale parameter. The corresponding reparameterization parameters$\gamma$=$Q^{-2}$, $u=\gamma\exp(|Q|z)$, $z=sign(Q)\frac{log(t)-\alpha}{\eta}$. When the effect size depends only on the location parameter, the parameterization of the hazard function for q versus b is as follows:

$$\log(t_{\mathrm{ij}})=\alpha x_{\mathrm{ij}}$$

where,$x_{\mathrm{ij}}$ is an exponential variable, which takes 0 when the patient i receives b in trial j and 1 when receiving q.

The second step is to estimate the mean and confidence interval of the effect size, denoted as:

$$\hat{\alpha}_{j,bq}\sim N(\alpha_{j,bq},Var(\hat{\alpha}_{j,bq}))$$

$Var(\hat{\alpha}_{j,bq})$ are variances; $\alpha_{j,bq}$ subject to the uninformative Normal prior distribution with a mean of 0 and a standard error of 0.001.

1. Restrictive mean survival time

RMST is the area under the curve of the survival curve in a specific time period, and the larger the area under the curve means the better efficacy. RMST can be regarded as a surrogate index of HR. The difference between the RMST of the two groups can more accurately reflect the difference in the efficacy of the treatment programs, and it also avoids the PH assumption and other problems. A two-step approach can be used to fit the RMST model. In the first step, the non-parametric KM method is used to estimate the total survival time, and the RMST ($\psi_{t}$) in the t period is calculated as follows:

$$\psi_{\mathrm{jq}}=\int_{0}^{t} \hat{S}(t)dt$$

j and q represent the j trial and the q treatment, respectively. It is worth noting that t generally takes the minimum value of the longest study duration of all treatments. In the second step, the estimated mean and standard error of $\psi_{t}$ are introduced into the NMA model under the Bayesian framework to estimate the RMST difference ($\psi_{q}$) of each treatment q compared to the standard treatment (q=1). Taking the fixed effects model as an example, the fitting process is as follows:

$$\hat{\psi}_{\mathrm{jq}}\sim N(\psi_{\mathrm{jq}},Var(\hat{\psi}_{\mathrm{jq}}))$$

$$\hat{\psi}_{\mathrm{jq}}=\left\{ \begin{aligned} \mu_{j} if q=1 \\ \mu_{j}+\psi_{q} if q\neq1 \end{aligned} \right.$$

$\mu_{j}$ represents the total survival time of the reference program, $\psi_{q}$ is the RMST difference between the q and the reference treatment. $\psi_{\mathrm{jq}}$ obeys an uninformative normal prior distribution with a mean of 0 and a standard error of 0.0001.

1. Fractional Polynomial Model

Fractional polynomial (FP) models were proposed by Jansen et al., includes the first-order and second-order FP. Among them, p1 and p2 can take any value in (-2, -1, -0.5, 0, 0.5, 1, 2, 3). Therefore, there are a total of 8 first-order FP models and 36 second-order FP models. In the first-order FP model, when p=0, the FP model is consistent with the Weibull model; when p=1, the FP model is equivalent to the Gompertz model. The risk equation of the FP model is as follows:

$$\left\{ \begin{aligned} \ln(h_{jqt})=\beta_{0,jq}+\beta_{1,jq}t^{p}, with t^{0}=log(t) 1-order FP \\ \ln(h_{jqt})=\beta_{0,jq}+\beta_{1,jq}t^{p_{1}}+\beta_{2,jq}t^{p_{2}}, with t^{0}=log(t) 2-order FP \end{aligned} \right.$$

$$\left\{ \begin{aligned} \left( \begin{matrix} \beta_{0,jq} \\ \beta_{1,jq} \end{matrix} \right)=\left( \begin{matrix} \alpha_{0,jq} \\ \alpha_{1,jq} \end{matrix} \right)+\left( \begin{matrix} d_{0,jq} \\ d_{1,jq} \end{matrix} \right) 1-order FP \\ \left( \begin{matrix} \beta_{0,jq} \\ \beta_{1,jq} \\ \beta_{2,jq} \end{matrix} \right)=\left( \begin{matrix} \alpha_{0,jq} \\ \alpha_{1,jq} \\ \alpha_{2,jq} \end{matrix} \right)+\left( \begin{matrix} d_{0,jq} \\ d_{1,jq} \\ d_{2,jq} \end{matrix} \right) 2-order FP \end{aligned} \right.$$

$h_{jqt}$ represents the risk value of the q treatment in the j trial at time t; $\alpha_{i,jq}$ (i can take from 0~2) indicates the baseline risk value of the control treatment; $d_{i,jq}$(i can take from 0~2) indicates the differences between the risk values of each treatment relative to the control treatment, $\beta_{i,jq}$ (i can take from 0~2) is the final risk value of each treatment. In the Bayesian fixed-effect model, the sum is subject to an uninformative normal prior distribution. Taking the first-order FP model as an example, its mean and standard error are $\binom{0}{0}$ and $\left( \begin{matrix} 0.001 & 0 \\ 0 & 0.001 \end{matrix} \right)$, respectively.

1. Royston-Parmar model

Royston-Parmar (RP) models can be divided into different intervals by nodes, and independent models are used to fit the data in these intervals. The RCS model, that is, the restricted cubic spline model, is based on the RP model and requires that the two intervals at both ends of the independent variable range be linear functions. The RCS model can be expressed as follows:

$$S_{j}(ln(t_{i}))=\gamma_{1}+\gamma_{2}u_{0}(ln(t_{i}))+...+\gamma_{p+2}u_{p}(ln(t_{i}))$$

$ln(t_{i})$ is the time at which the patient i's event occurred, $p$ representing the number of nodes, which will lead to $p+2$ periods; $u_{0\sim p}(ln(t_{i}))$ representing the orthogonalized basic equation of each node, $\gamma$ representing the regression coefficient.

The basic equation is as follows:

$$v_{m}(ln(t_{i}))={{(ln(t_{i})-k_{m})}_{+}}^{3}-\lambda_{m}{{(ln(t_{i})-k_{0})}_{+}}^{3}-\lambda_{m}{{(ln(t_{i})-k_{p+1})}_{+}}^{3}$$

$$\lambda_{m}=\frac{k_{p+1}-k_{m}}{k_{p+1}-k_{0}}$$

Among them, $m$ can take $\mathrm{from}0\sim p$, ${(x)}_{+}$ is the indicator function. When x takes 0, ${(x)}_{+}$ equal x, otherwise equal to 0, $k_{0\sim p+1}$ is the time point of the node.

The Gram-Schmidt orthogonal method is used to make $v_{m}$ converted to $u_{m}$. Assume $u_{m}(ln(t_{i}))=u_{\mathrm{nm}}$, the process is as follows:

$$\ln\left\{ H_{j}\left( t | \mathrm{trt}_{\mathrm{qi}} \right) \right\}=S_{j}(ln(t_{i}))+\beta_{1}\mathrm{trt}_{1i}+...+\beta_{q}\mathrm{trt}_{\mathrm{qi}}+\beta_{q+1}\mathrm{trt}_{1i}(ln(t_{i}))+...\beta_{2q}\mathrm{trt}_{\mathrm{qi}}(ln(t_{i}))$$

$\mathrm{trt}_{1\sim q}$ represents q treatments, $\beta_{1\sim q}$are scale parameters and $\beta_{q+1\sim2q}$are shape parameters. Under the Bayesian fixed-effect model, $\beta$ obeys the Normal prior distribution with no prior information, and the mean and standard error are 0 and 0.0001, respectively.

# Table S1 Details of search strategy

| **MEDLINE** | |
| --- | --- |
| **#1** | ((Carcinoma, Non Small Cell Lung OR Carcinomas, Non-Small-Cell Lung OR Lung Carcinoma, Non-Small-Cell OR Lung Carcinomas, Non-Small-Cell OR Non-Small-Cell Lung Carcinomas OR Non-Small-Cell Lung Carcinoma OR Non Small Cell Lung Carcinoma OR Carcinoma, Non-Small Cell Lung OR Non-Small Cell Lung Carcinoma OR Non-Small Cell Lung Cancer OR Nonsmall Cell Lung Cancer) AND (Non-squamous OR Nonsquamous)):ti,ab,kw |
| **#2** | (Checkpoint Inhibitors, Immune OR Immune Checkpoint Inhibitor OR Checkpoint Inhibitor, Immune OR Immune Checkpoint Blockers OR Checkpoint Blockers, Immune OR Immune Checkpoint Blockade OR Checkpoint Blockade, Immune OR Immune Checkpoint Inhibition OR Checkpoint Inhibition, Immune OR PD-L1 Inhibitors OR PD L1 Inhibitors OR PD-L1 Inhibitor OR PD L1 Inhibitor OR Programmed Death-Ligand 1 Inhibitors OR Programmed Death Ligand 1 Inhibitors OR PD-1-PD-L1 Blockade OR Blockade, PD-1-PD-L1 OR PD 1 PD L1 Blockade OR PD-1 Inhibitors OR PD 1 Inhibitors OR PD-1 Inhibitor OR Inhibitor, PD-1 OR PD 1 Inhibitor OR Programmed Cell Death Protein 1 Inhibitor OR Programmed Cell Death Protein 1 Inhibitors OR Sintilimab OR Camrelizumab OR Atezolizumab OR Pembrolizumab OR Sugemalimab):ti,ab,kw |
| **#3** | (randomized controlled trial OR clinical trial OR phase III OR phase 3):ti,ab,kw |
| **#4** | #1 AND #2 AND #3 |
| **Embase** | |
| **#1** | ((Carcinoma, Non Small Cell Lung OR Carcinomas, Non-Small-Cell Lung OR Lung Carcinoma, Non-Small-Cell OR Lung Carcinomas, Non-Small-Cell OR Non-Small-Cell Lung Carcinomas OR Non-Small-Cell Lung Carcinoma OR Non Small Cell Lung Carcinoma OR Carcinoma, Non-Small Cell Lung OR Non-Small Cell Lung Carcinoma OR Non-Small Cell Lung Cancer OR Nonsmall Cell Lung Cancer) AND (Non-squamous OR Nonsquamous)):ti,ab,kw |
| **#2** | (Checkpoint Inhibitors, Immune OR Immune Checkpoint Inhibitor OR Checkpoint Inhibitor, Immune OR Immune Checkpoint Blockers OR Checkpoint Blockers, Immune OR Immune Checkpoint Blockade OR Checkpoint Blockade, Immune OR Immune Checkpoint Inhibition OR Checkpoint Inhibition, Immune OR PD-L1 Inhibitors OR PD L1 Inhibitors OR PD-L1 Inhibitor OR PD L1 Inhibitor OR Programmed Death-Ligand 1 Inhibitors OR Programmed Death Ligand 1 Inhibitors OR PD-1-PD-L1 Blockade OR Blockade, PD-1-PD-L1 OR PD 1 PD L1 Blockade OR PD-1 Inhibitors OR PD 1 Inhibitors OR PD-1 Inhibitor OR Inhibitor, PD-1 OR PD 1 Inhibitor OR Programmed Cell Death Protein 1 Inhibitor OR Programmed Cell Death Protein 1 Inhibitors OR Sintilimab OR Camrelizumab OR Atezolizumab OR Pembrolizumab OR Sugemalimab):ti,ab,kw |
| **#3** | (randomized controlled trial OR clinical trial OR phase III OR phase 3):ti,ab,kw |
| **#4** | #1 AND #2 AND #3 |
| **Cochrane Central Register of Controlled Trials (CENTRAL)** | |
| **#1** | ((Carcinoma, Non Small Cell Lung OR Carcinomas, Non-Small-Cell Lung OR Lung Carcinoma, Non-Small-Cell OR Lung Carcinomas, Non-Small-Cell OR Non-Small-Cell Lung Carcinomas OR Non-Small-Cell Lung Carcinoma OR Non Small Cell Lung Carcinoma OR Carcinoma, Non-Small Cell Lung OR Non-Small Cell Lung Carcinoma OR Non-Small Cell Lung Cancer OR Nonsmall Cell Lung Cancer) AND (Non-squamous OR Nonsquamous)):ti,ab,kw |
| **#2** | (Checkpoint Inhibitors, Immune OR Immune Checkpoint Inhibitor OR Checkpoint Inhibitor, Immune OR Immune Checkpoint Blockers OR Checkpoint Blockers, Immune OR Immune Checkpoint Blockade OR Checkpoint Blockade, Immune OR Immune Checkpoint Inhibition OR Checkpoint Inhibition, Immune OR PD-L1 Inhibitors OR PD L1 Inhibitors OR PD-L1 Inhibitor OR PD L1 Inhibitor OR Programmed Death-Ligand 1 Inhibitors OR Programmed Death Ligand 1 Inhibitors OR PD 1 PD L1 Blockade OR PD-1 Inhibitors OR PD 1 Inhibitors OR PD-1 Inhibitor OR Inhibitor, PD-1 OR PD 1 Inhibitor OR Programmed Cell Death Protein 1 Inhibitor OR Programmed Cell Death Protein 1 Inhibitors OR Sintilimab OR Camrelizumab OR Atezolizumab OR Pembrolizumab OR Sugemalimab):ti,ab,kw |
| **#3** | (randomized controlled trial OR clinical trial OR phase III OR phase 3):ti,ab,kw |
| **#4** | #1 AND #2 AND #3 |
| **Web of Science** | |
| **#1** | TS=((Carcinoma, Non Small Cell Lung OR Carcinomas, Non-Small-Cell Lung OR Lung Carcinoma, Non-Small-Cell OR Lung Carcinomas, Non-Small-Cell OR Non-Small-Cell Lung Carcinomas OR Non-Small-Cell Lung Carcinoma OR Non Small Cell Lung Carcinoma OR Carcinoma, Non-Small Cell Lung OR Non-Small Cell Lung Carcinoma OR Non-Small Cell Lung Cancer OR Nonsmall Cell Lung Cancer) AND (Non-squamous OR Nonsquamous)) |
| **#2** | TS=(Checkpoint Inhibitors, Immune OR Immune Checkpoint Inhibitor OR Checkpoint Inhibitor, Immune OR Immune Checkpoint Blockers OR Checkpoint Blockers, Immune OR Immune Checkpoint Blockade OR Checkpoint Blockade, Immune OR Immune Checkpoint Inhibition OR Checkpoint Inhibition, Immune OR PD-L1 Inhibitors OR PD L1 Inhibitors OR PD-L1 Inhibitor OR PD L1 Inhibitor OR Programmed Death-Ligand 1 Inhibitors OR Programmed Death Ligand 1 Inhibitors OR PD-1-PD-L1 Blockade OR Blockade, PD-1-PD-L1 OR PD 1 PD L1 Blockade OR PD-1 Inhibitors OR PD 1 Inhibitors OR PD-1 Inhibitor OR Inhibitor, PD-1 OR PD 1 Inhibitor OR Programmed Cell Death Protein 1 Inhibitor OR Programmed Cell Death Protein 1 Inhibitors OR Sintilimab OR Camrelizumab OR Atezolizumab OR Pembrolizumab OR Sugemalimab) |
| **#3** | TS=(randomized controlled trial OR clinical trial OR phase III OR phase 3) |
| **#4** | #1 AND #2 AND #3 |

# Table S2 Basic information of included randomized controlled trials

| **Trial title** | **Group** | **Indica-tions** | **Patient source** | **Medications and therapies** | **Sample size** | **Average age** | **Proportion of males (%)** | **ECOG PS** | **Disease stage** | **Smoking**  **proportion** | **HR(PFS)** | **HR(OS)** | **Median PFS (month)** | **Median OS (month)** | **Objective response rate** |
| --- | --- | --- | --- | --- | --- | --- | --- | --- | --- | --- | --- | --- | --- | --- | --- |
| ORIENT-11 | Treatment group | Locally advanced or metastatic nsq-NSCLC | China | Sintilimab  +  chemotherapy | 266 | 61 | 77 | 1:71% | III : 8%; IV : 92% | 64% | 0.48  (0.36-0.64) | 0.61  (0.40-0.93) | 8.9  (7.1-11.3) | NR | 51.9% |
|  | Control group |  |  | Chemotherapy | 131 | 61 | 76 | 1:74% | III : 11%; IV : 89% | 66% |  |  | 5.0  (4.8-6.2) | NR | 29.8% |
| CamL | Treatment group | Advanced nsq-NSCLC | China | Camrelizumab  +  chemotherapy | 205 | 59 | 71 | 1:77% | III : 15%; IV : 85% | 62% | 0.60  (0.45-0.79) | 0.73  (0.53-1.02) | 11.3  (9.6-15.4) | NR  (16.6-NR) | 60.5% |
|  | Control group |  |  | Chemotherapy | 207 | 61 | 72 | 1:83% | III : 20%; IV : 80% | 63% |  |  | 8.3  (6.0-9.7) | 20.9  (14.2-NR) | 38.6% |
| IMpower  132 | Treatment group | Advanced  nsq-NSCLC | Global | Atezolizumab  +  chemotherapy | 292 | 64 | 66 | 1:57% | IV : 100% | 87% | 0.60  (0.49-0.72) | 0.86  (0.71-1.06) | 7.6  (6.6-8.5) | 17.5  (13.2-19.6) | 47% |
|  | Control group |  |  | Chemotherapy | 286 | 63 | 67 | 1:60% | IV : 100% | 90% |  |  | 5.2  (4.3-5.6) | 13.6  (11.0-15.7) | 32% |
| KEYNOTE-  189 | Treatment group | Advanced nsq-NSCLC | Global | Pembrolizumab  +  chemotherapy | 410 | 65 | 62 | 1:54% | IV : 100% | 88% | 0.49  (0.41-0.59) | 0.56  (0.46-0.69) | 9.0  (8.1-10.4) | 22.0  (19.5-24.5) | 48.3% |
|  | Control group |  |  | Chemotherapy | 206 | 64 | 53 | 1:61% | IV : 100% | 88% |  |  | 4.9  (4.7-5.5) | 10.6  (8.7-13.6) | 19.9% |
| GEMSTONE-  302 | Treatment group | Advanced nsq-NSCLC | China | Sugemalimab  +  chemotherapy | 320 | 62 | 79 | 1:82% | IV : 100% | 73% | 0.59  (0.45-0.79) | 0.84  (0.57-1.23) | 9.6  (8.3-11.0) | 22.8 | 63.4% |
|  | Control group |  |  | Chemotherapy | 159 | 64 | 81 | 1:84% | IV : 100% | 75% |  |  | 5.9  (4.9-7.1) | 20.1 | 40.3% |

Abbreviations: nsq-NSCLC, non-squamous non-small cell lung cancer; HR, hazard ratio; PFS, progression-free survival; OS, overall survival; ECOG PS, Eastern Cooperative Oncology Group Performance Status; NR, not reported

# Table S3 Descriptions of treatment regimens

| **Clinical Trial** | **Interventions** | **Drug Regimen** | **Reference** |
| --- | --- | --- | --- |
| ORIENT-11 | Sintilimab  +  chemotherapy | Induction therapy administered every cycle includes sintilimab (200mg, day1), pemetrexed (500mg/m^2^), cisplatin (75mg/m^2^, day1) or carboplatin (AUC 5 mg/mL/min, day1) and lasts for 4 or 6 cycles, followed by sintilimab and pemetrexed for up to 2 years until disease progression or related serious adverse events. | (1) |
|  | chemotherapy | Induction therapy administered every cycle includes placebo (200mg, day1), pemetrexed (500mg/m^2^) and cisplatin (75mg/m^2^, day1) or carboplatin (AUC 5 mg/mL/min, day1) and lasts for 4 or 6 cycles, followed by placebo and pemetrexed for up to 2 years until disease progression or related serious adverse events. | (1) |
| CameL | Camrelizumab  +  chemotherapy | Induction therapy administered every cycle includes camrelizumab (1200mg, day1), pemetrexed (500mg/m^2^, day1), carboplatin (AUC 5 mg/mL/min, day1) and lasts for 4 or 6 cycles, followed by camrelizumab and pemetrexed for up to 2 years until disease progression or related serious adverse events. | (2) |
|  | chemotherapy | Induction therapy administered every cycle includes placebo (1200mg, day1), pemetrexed (500mg/m^2^, day1), carboplatin (AUC 5 mg/mL/min, day1) and lasts for 4 or 6 cycles, followed by placebo and pemetrexed for up to 2 years until disease progression or related serious adverse events. | (2) |
| IMpower132 | Atezolizumab  +  chemotherapy | Induction therapy administered every cycle includes atezolizumab (1200mg, day1), cisplatin (75mg/m^2^, day1) or carboplatin (AUC 6 mg/mL/min, day1) and pemetrexed (500mg/m^2^) and lasts for 4 or 6 cycles, followed by atezolizumab and pemetrexed for up to 2 years until disease progression or related serious adverse events. | (3) |
|  | Chemotherapy | Induction therapy administered every cycle includes placebo (1200mg, day1), cisplatin (75mg/m^2^, day1) or carboplatin (AUC 6 mg/mL/min, day1) and pemetrexed (500mg/m^2^) and lasts for 4 or 6 cycles, followed by placebo and pemetrexed for up to 2 years until disease progression or related serious adverse events. | (3) |
| KEYNOTE-189 | Pembrolizumab  +  chemotherapy | Induction therapy administered every cycle includes pembrolizumab (200mg, day1), cisplatin (75mg/m^2^, day1) or carboplatin (AUC 5 mg/mL/min, day1) and pemetrexed (500mg/m^2^) and lasts for 4 cycles, followed by pembrolizumab and pemetrexed for up to 2 years until disease progression or related serious adverse events. | (4) |
|  | Chemotherapy | Induction therapy administered every cycle includes placebo (200mg, day1), cisplatin (75mg/m^2^, day1) or carboplatin (AUC 5 mg/mL/min, day1) and pemetrexed (500mg/m^2^) and lasts for 4 cycles, followed by placebo and pemetrexed for up to 2 years until disease progression or related serious adverse events. | (4) |
| GEMSTONE-302 | Sugemalimab  +  chemotherapy | Induction therapy administered every cycle includes sugemalimab (1200mg, day1), pemetrexed (500mg/ m^2^, day1) and carboplatin (AUC 5 mg/mL/min, day1) and lasts for 4 cycles, followed by sugemalimab and pemetrexed for up to 2 years until disease progression or related serious adverse events. | (5) |
|  | Chemotherapy | Induction therapy administered every cycle includes placebo (1200mg, day1), pemetrexed (500mg/ m^2^, day1) and carboplatin (AUC 5 mg/mL/min, day1) and lasts for 4 cycles, followed by placebo and pemetrexed for up to 2 years until disease progression or related serious adverse events. | (5) |

# Table S4 The proportion of patients receiving follow-up active treatment and best supportive care in subsequent treatment

| **First-line treatment** | **Active therapy** | **Best supportive care** | **Reference** |
| --- | --- | --- | --- |
| Sintilimab+chemotherapy | 0.476 | 0.524 | Assumed |
| Camrelizumab+chemotherapy | 0.420 | 0.580 | (2) |
| Atezolizumab+chemotherapy | 0.613 | 0.387 | (3) |
| Pembrolizumab+chemotherapy | 0.495 | 0.505 | (4) |
| Sugemalimab+chemotherapy | 0.559 | 0.441 | (5) |

# Table S5 Summary of disease management costs and follow-up costs

| **Medical resource type** | **Unit cost (range)/$** | **Resource utilization (time/every 3 weeks)** |
| --- | --- | --- |
| Disease management | | |
| Diagnosis | 2.95 (1.36~4.08) | 1 |
| PD-L1 test | 48.50 (38.80~58.20) | 1(during the first cycle) |
| Nursing | 4.22 (3.52~4.92) | 3 |
| Intravenous injection | 1.41 (1.36~1.88) | PFS: 3; PD: 2 |
| Bed | 7.03 (4.57~9.14) | 3 |
| Follow-up | | |
| CT | 50.63 (40.30~60.44) | PFS: 1-4 cycles: 0.5; 5~12 cycles: 1/3; after 12 cycles: 0.25; PD: 0.25 |
| Routine blood test | 2.81 (2.18~3.27) | PFS:1; PD: 0.75 |
| Blood chemistry examination | 36.29 (32.59~48.89) | PFS:1; PD: 0.75 |
| Routine urine test | 299.29 (236.62~354.93) | PFS:1; PD: 0.75 |
| End-of-life care | 2298.86 (892.71~6140.16) | 1 (3 months before death) |

# Table S6 Information of patient assistant programs

| **Drug** | **Condition** | **Patient assistant scheme** |
| --- | --- | --- |
| Atezolizumab | In combination with pemetrexed and platinum-based chemotherapy for first-line treatment of epidermal growth factor receptor (EGFR) mutation-negative and anaplastic lymphoma kinase (ALK)-negative metastatic NSCLC | In the first round, patients receive 3 cycles of assistance after purchasing 2 cycles of treatment; in the second round, patients receive continuous assistance after purchasing 2 cycles of treatment for up to 1 year |
| Pembrolizumab | In combination with pemetrexed and platinum-based chemotherapy for first-line treatment of EGFR mutation-negative and ALK-negative metastatic NSCLC | In the first round, patients receive 2 cycles of assistance after purchasing 2 cycles of treatment; in the second round, patients receive continuous assistance after purchasing 2 cycles of treatment for up to 2 years |
| Sugemalimab | In combination with pemetrexed and carboplatin for first-line treatment of patients with EGFR mutation-negative and ALK-negative metastatic | In the first round, patients receive 2 cycles of assistance after purchasing 2 cycles of treatment; in the second round, patients receive 25 cycles of assistance after purchasing 2 cycles of treatment; in the third round, patients receive 3 cycles of assistance after purchasing 1 cycle of treatment |

# Table S7 The goodness-of-fit results of various models

| **RP model for PFS** | | | **RP model for OS** | | |
| --- | --- | --- | --- | --- | --- |
| **K** | **Scale** | **AIC** | **K** | **Scale** | **AIC** |
| 0 | hazard | 219.52 | 0 | hazard | 567.52 |
|  | odds | 207.41 |  | odds | 564.26 |
|  | normal | 206.60 |  | normal | 575.27 |
| 1 | hazard | 206.59 | 1 | hazard | 567.88 |
|  | odds | 209.41 |  | odds | 564.68 |
|  | normal | 206.97 |  | normal | 565.45 |
| 2 | hazard | 208.27 | 2 | hazard | 562.92 |
|  | odds | 209.17 |  | odds | 563.92 |
|  | normal | 208.97 |  | normal | 564.09 |
| 3 | hazard | 210.14 | 3 | hazard | 562.92 |
|  | odds | 211.03 |  | odds | 563.92 |
|  | normal | 210.94 |  | normal | 564.09 |
| 4 | hazard | 211.29 | 4 | hazard | 565.34 |
|  | odds | 211.92 |  | odds | 566.32 |
|  | normal | 212.61 |  | normal | 566.31 |
| 5 | hazard | 213.52 | 5 | hazard | 565.49 |
|  | odds | 214.09 |  | odds | 565.84 |
|  | normal | 214.38 |  | normal | 565.02 |
| **Standard parametric model for PFS** | | | **Standard parametric model for OS** | | |
| **Distribuion** | | **AIC** | **Distribuion** | | **AIC** |
| Exponential | | 238.24 | Exponential | | 632.47 |
| Weibull | | 219.52 | Weibull | | 634.27 |
| Gamma | | 213.27 | Gamma | | 634.47 |
| Log-normal | | 206.60 | Log-normal | | 629.29 |
| Gompertz | | 234.36 | Gompertz | | 630.33 |
| Log-logistic | | 207.41 | Log-logistic | | 624.22 |
| Generalized gamma | | 206.71 | Generalized gamma | | 628.08 |
| Generalized F | | 208.71 | Generalized F | | 627.20 |

# Table S8 AIC values of various FP models

| **Value of parameter p** | **AIC** |
| --- | --- |
| -2 | 1028.15 |
| -1 | 1028.94 |
| -0.5 | 1029.07 |
| 0 | 1028.48 |
| 0.5 | 1026.96 |
| 1 | 1024.95 |
| 2 | 1021.79 |
| 3 | 1021.08 |


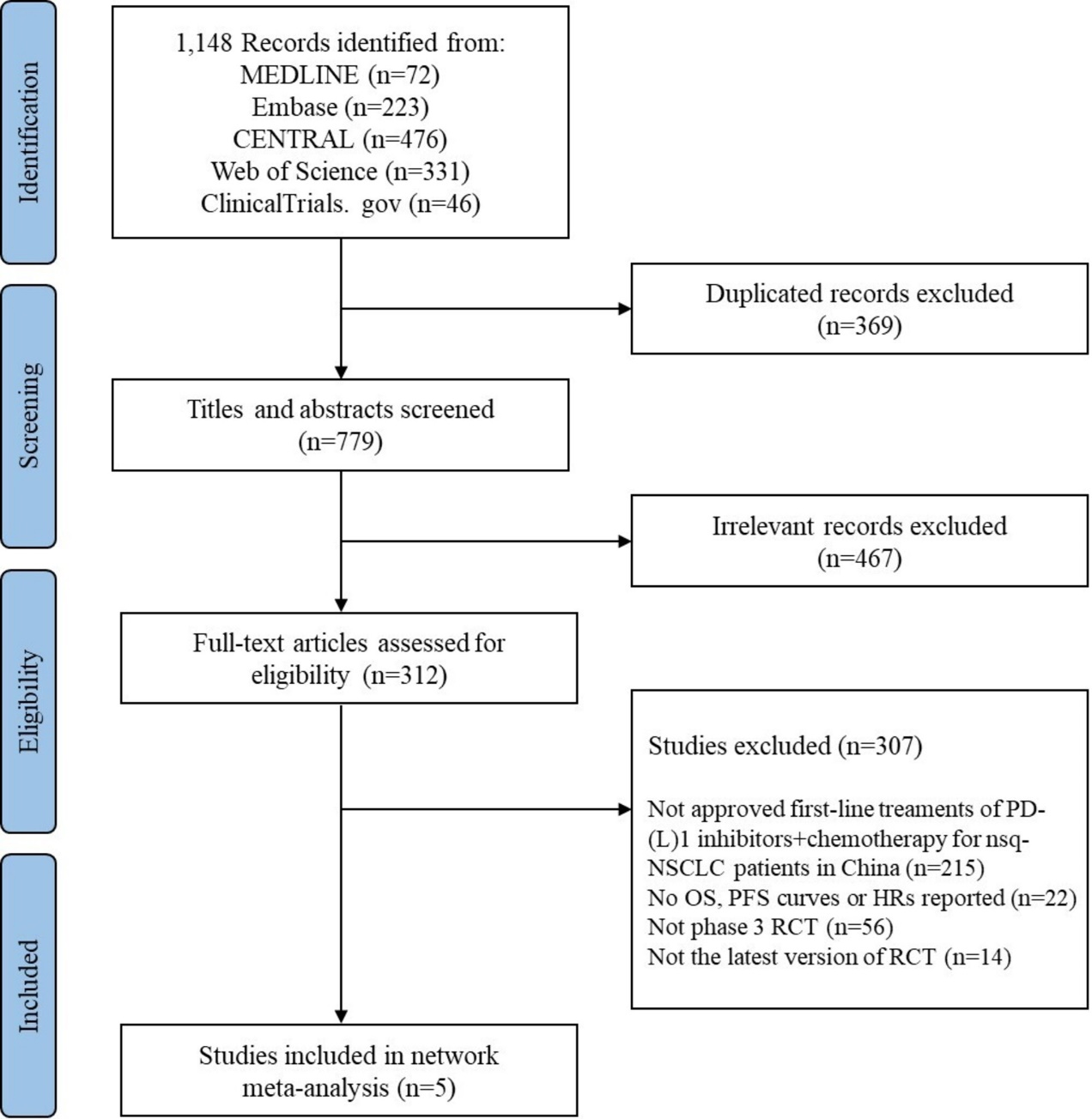


# Figure S1 The PRISMA flow diagram


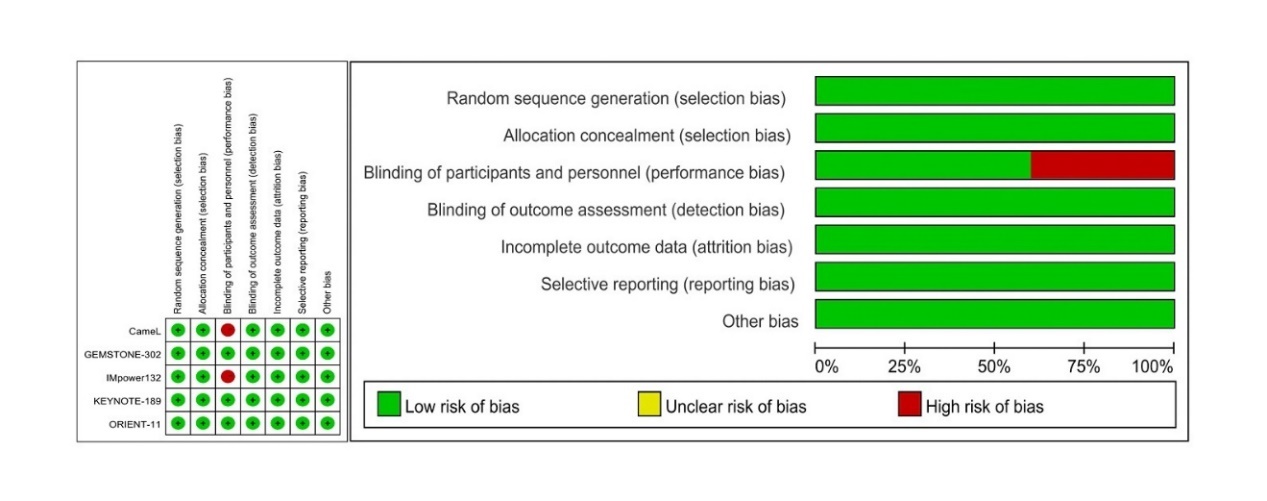


# Figure S2 Risk of bias assessment outcome


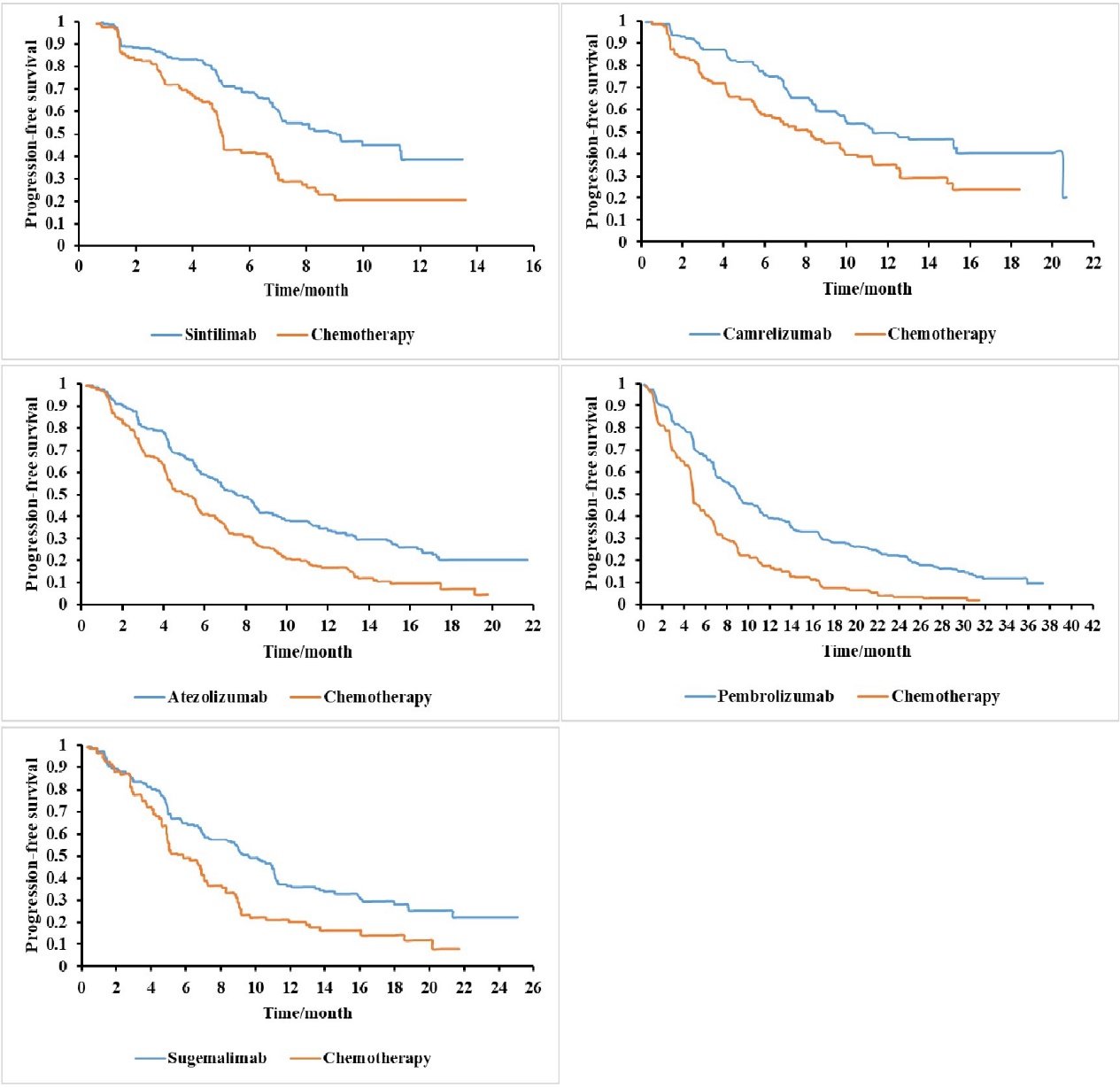


# Figure S3 Original Kaplan-Meier plots of progression-free survival


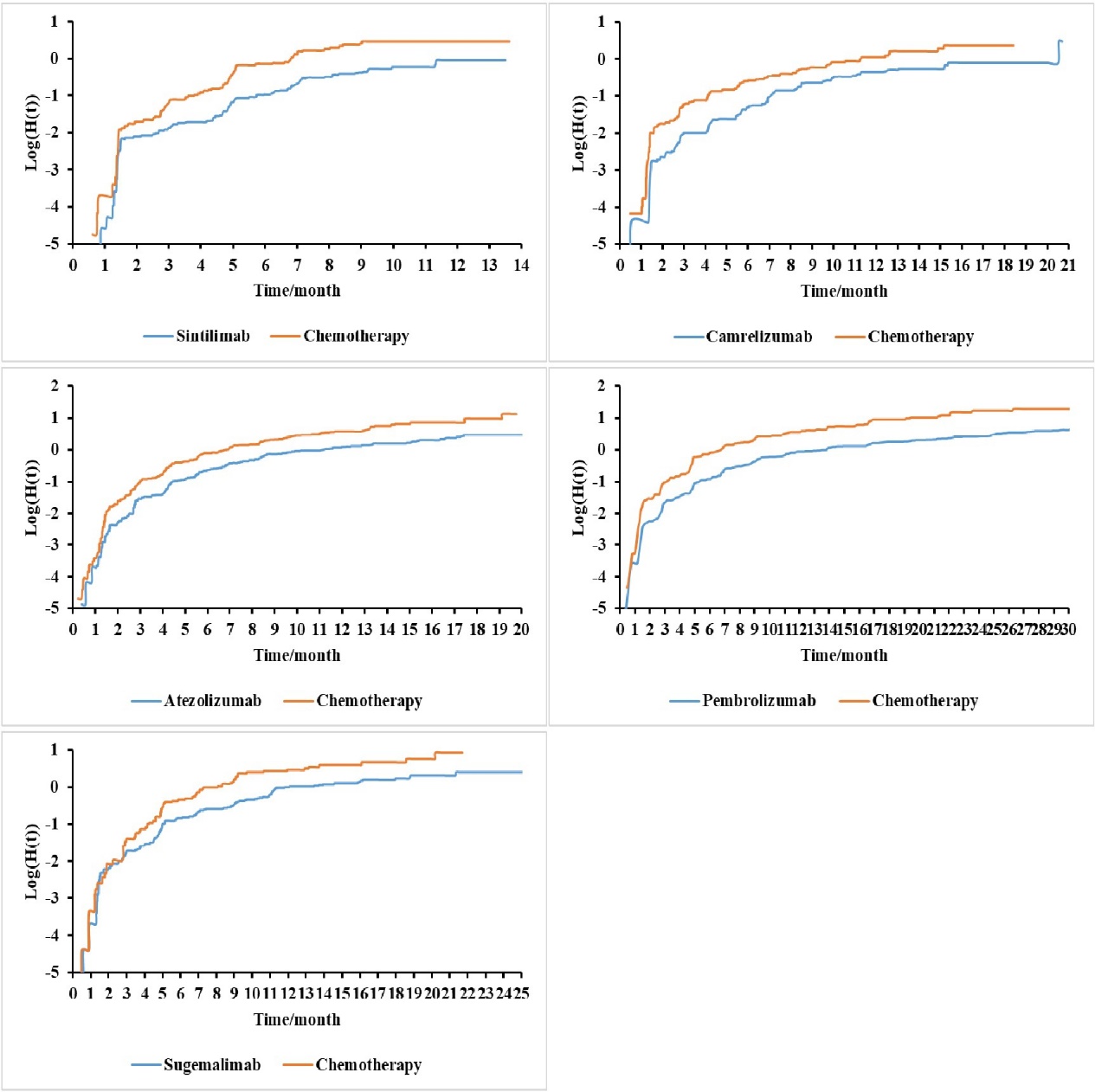


# Figure S4 Log-cummulative hazard plots of the progression-free survival of pateints diagnosed with nsq-NSCLC

|  |
| --- |
| 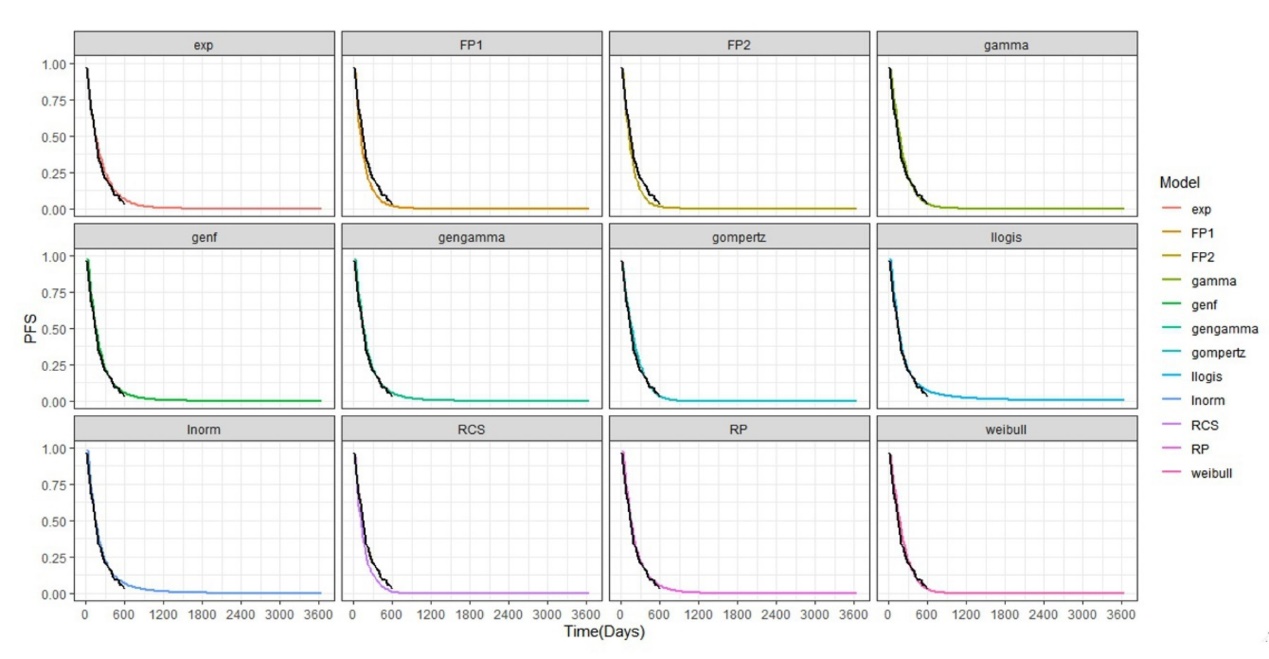 |

# Figure S5 The fitting performance of all candidate models for progression-free survival


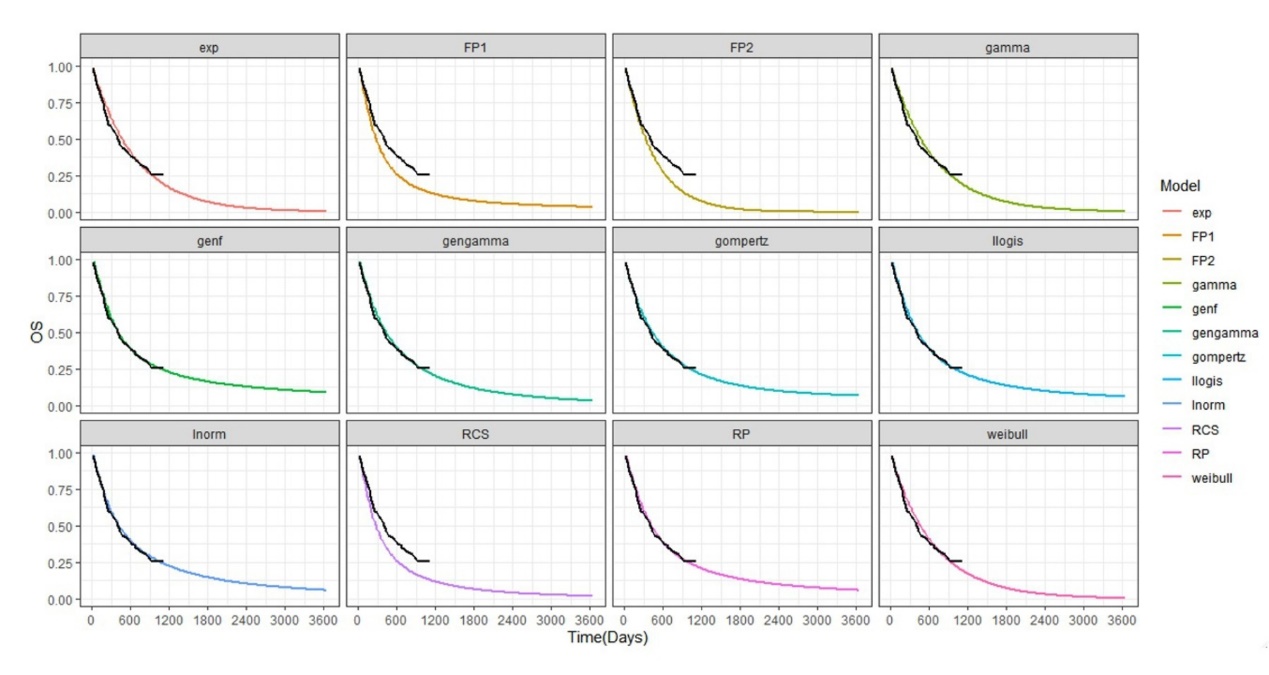


# Figure S6 The fitting performance of all candidate models for overall survival


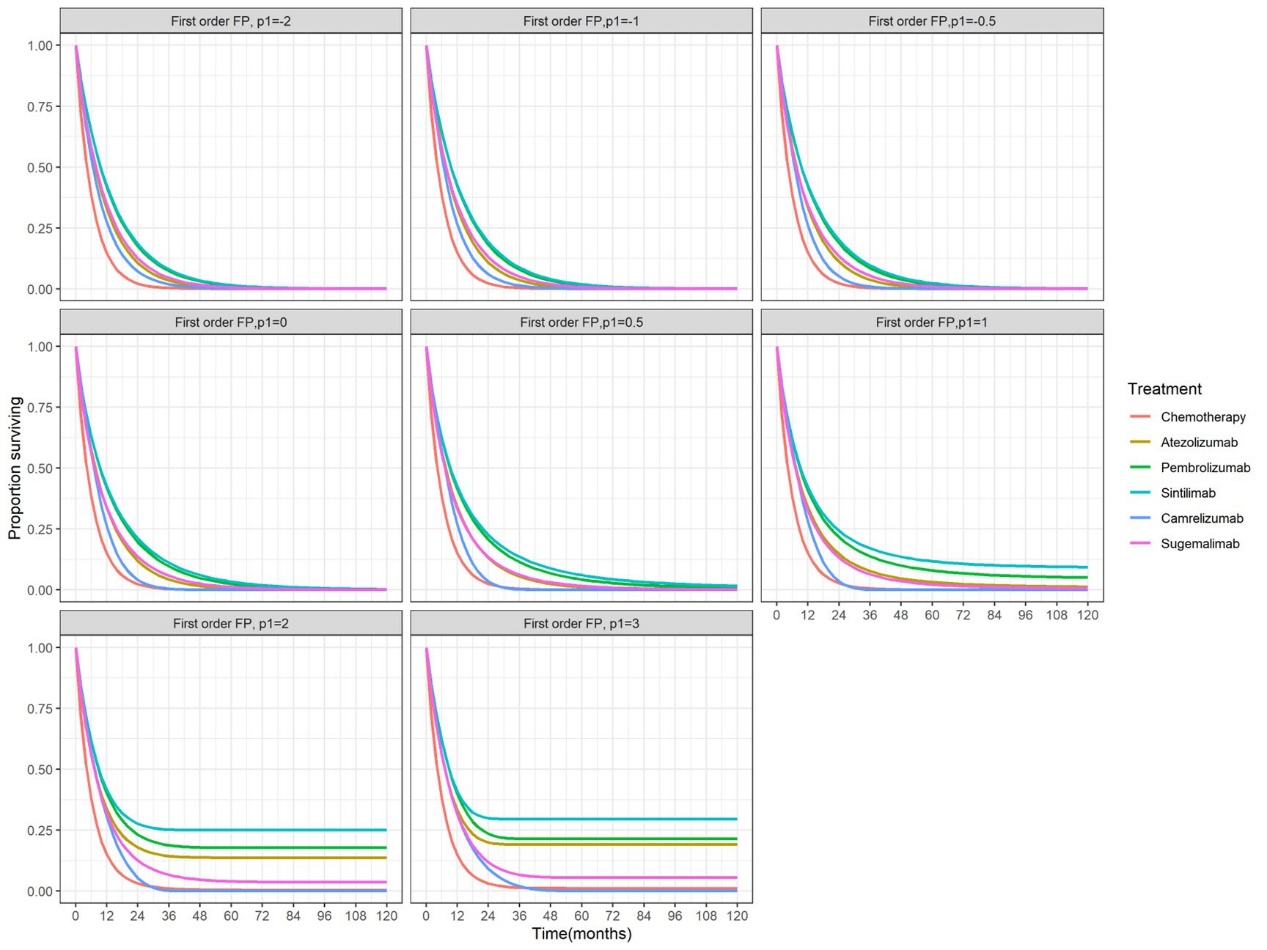


# Figure S7 Fitted curves of all FP models

# Reference

1. Yang Y, Wang Z, Fang J, et al. Efficacy and Safety of Sintilimab Plus Pemetrexed and Platinum as First-Line Treatment for Locally Advanced or Metastatic Nonsquamous NSCLC: a Randomized, Double-Blind, Phase 3 Study (Oncology pRogram by InnovENT anti-PD-1-11). *J Thorac Oncol*. (2020) 15(10):1636-1646. doi: 10.1016/j.jtho.2020.07.014
2. Zhou C, Chen G, Huang Y, et al. Camrelizumab plus carboplatin and pemetrexed versus chemotherapy alone in chemotherapy-naive patients with advanced non-squamous non-small-cell lung cancer (CameL): a randomised, open-label, multicentre, phase 3 trial. *Lancet Respir Med*. (2021) 9(3):305-314. doi: 10.1016/S2213-2600(20)30365-9
3. Nishio M, Barlesi F, West H, et al. Atezolizumab Plus Chemotherapy for First-Line Treatment of Nonsquamous NSCLC: Results From the Randomized Phase 3 IMpower132 Trial. *J Thorac Oncol*. (2021) 16(4):653-664. doi: 10.1016/j.jtho.2020.11.025
4. Rodríguez-Abreu D, Powell SF, Hochmair MJ, et al. Pemetrexed plus platinum with or without pembrolizumab in patients with previously untreated metastatic nonsquamous NSCLC: protocol-specified final analysis from KEYNOTE-189. *Ann Oncol*. (2021) 32(7):881-895. doi: 10.1016/j.annonc.2021.04.008
5. Zhou C, Wang Z, Sun Y, et al. Sugemalimab versus placebo, in combination with platinum-based chemotherapy, as first-line treatment of metastatic non-small-cell lung cancer (GEMSTONE-302): interim and final analyses of a double-blind, randomised, phase 3 clinical trial. *Lancet Oncol*. (2022) 23(2):220-233. doi: 10.1016/S1470-2045(21)00650-1
